# Partitioned dual Maclaurin symmetric mean operators based on picture fuzzy sets and their applications in multi-attribute decision-making problems

**DOI:** 10.1038/s41598-023-44344-8

**Published:** 2023-11-27

**Authors:** Tahir Mahmood, Ubaid ur Rehman, Walid Emam, Zeeshan Ali, Haolun Wang

**Affiliations:** 1 Department of Mathematics and Statistics, International Islamic University Islamabad, Islamabad, Pakistan; 2 Department of Statistics and Operations Research, Faculty of Science, King Saud University, P.O. Box 2455, 11451 Riyadh, Saudi Arabia; 3 Department of Mathematics and Statistics, Riphah International University Islamabad, Islamabad, Pakistan; 4 School of Economics and Management, Nanchang Hangkong University, Nanchang, 330063 China

**Keywords:** Applied mathematics, Computer science

## Abstract

The partitioned Dual Maclaurin symmetric mean (PDMSM) operator has the supremacy that can justify the interrelationship of distinct characteristics and there are a lot of exploration consequences for it. However, it has not been employed to manage “multi-attribute decision-making” (MADM) problems represented by picture fuzzy numbers. The basic inspiration of this identification is to develop the novel theory of picture fuzzy PDMSM operator, and weighted picture fuzzy PDMSM operator and to identify their important results (Idempotency, Monotonicity, and Boundedness). Further, to identify the best decision, every expert realized that they needed the best way to find the beneficial optimal using the proper decision-making procedure, for this, we diagnosed the MADM tool in the consideration of deliberated approaches based on PF information. Finally, to drive the characteristics of the invented work, several examples are utilized to test the manifest of the comparative analysis with various more existing theories, which is a fascinating and meaningful technique to deeply explain the features and exhibited of the proposed approaches.

## Introduction

The aim of the decision-making (DM) sciences is to identify the massive dominant decision from a group of expected ones. MADM is a critical and crucial component of these sciences. In genuine DM strategy, the dilemma requires resolving the provided decisions by many classes like single or interval evaluation investigations. However, in many awkward scenarios, it is very challenging for the expert to reduce their decisions to a classical number. To establish it, Zadeh^1^ exposed the mathematical framework of fuzzy set (FS) by proposing a new function, called membership degree (MD)  defined from universal set $$X$$ to $$\left[0, 1\right]$$. If we are taking any arbitrary element $$x\in X$$ and assigning it to a value , then the resultant value should belong to $$\left[0, 1\right]$$. In many cases, the meaningful theory of FS has been unsuitable, when an expert copes with information that contains yes or no. Then for this, one of the most suitable and meaningful theories which easily manage awkward and complicated data arising in real life was invented by Atanassov^2, 3^, called intuitionistic FS (IFS) by including a new function, called non-membership degree (NMD) $$\eta_ {{\underset{\raise0.3em\hbox{$\smash{\scriptscriptstyle\cdot}$}}{\text{Y}} }}  $$ defined from universal set $$X$$ to $$\left[\mathrm{0,1}\right]$$. If we are taking any arbitrary element $$x \in X$$ and assigning it to a function $$\eta_{{\underset{\raise0.3em\hbox{$\smash{\scriptscriptstyle\cdot}$}}{\text{Y}} }}  \left( x \right)$$, then the resultant value should belong to $$\left[ {0,1} \right]$$ with $$0 \le \zeta_{{\underset{\raise0.3em\hbox{$\smash{\scriptscriptstyle\cdot}$}}{\text{Y}} }}  \left( x \right) + \eta_{{\underset{\raise0.3em\hbox{$\smash{\scriptscriptstyle\cdot}$}}{\text{Y}} }}  \left( x \right) \le 1$$. Unrelatedly, the informative idea of IFSs has been utilized in various areas. Still, there is a diversity of circumstances in which IFS theory can’t be employed. For instance, casting a vote and managing dilemmas like yes, abstinence, no, and refusal, which bounded the implementation of IFS theory. Then for this, one of the most suitable and meaningful theories which easily manage difficult and intricate data occurring in real life was invented by Cuong^4^, called picture FS (PFS) by including a new function, called abstinence degree (AD) $$\wp_{{\underset{\raise0.3em\hbox{$\smash{\scriptscriptstyle\cdot}$}}{\text{Y}} }} $$ defined from universal set $$X$$ to $$\left[\mathrm{0,1}\right]$$. If we are taking any arbitrary element $$x \in X$$ and assigning it to a function $$\overline{\wp }_ {{\underset{\raise0.3em\hbox{$\smash{\scriptscriptstyle\cdot}$}}{\text{Y}} }}\left( x \right)$$, then the resultant value should belong to $$\left[\mathrm{0,1}\right]$$ with $$0 \le \zeta_{{\underset{\raise0.3em\hbox{$\smash{\scriptscriptstyle\cdot}$}}{\text{Y}} }}  \left( x \right) + \wp_{{\underset{\raise0.3em\hbox{$\smash{\scriptscriptstyle\cdot}$}}{\text{Y}} }} \left( x \right) + \eta_{{\underset{\raise0.3em\hbox{$\smash{\scriptscriptstyle\cdot}$}}{{\underset{\raise0.3em\hbox{$\smash{\scriptscriptstyle\cdot}$}}{\text{Y}} }} }}  \left( x \right) \le 1$$.

### Literature review

Because of the importance and significance of the FS, numerous researchers worked in this area. Saha et al.^5^ devised a similarity-based arrangement with the Archimedean Dombi aggregation operator (AO) in the setting of fuzzy information. Babuska and Verbruggen^6^ deduced techniques for FS and Hullermeier^7^ employed techniques for FS in machine learning. The fuzzy technique of MADM was interpreted by Nazari et al.^8^. Deni et al.^9^ deduced another fuzzy technique of MADM and named it the SAW technique. The key concept of L-FS was diagnosed by Goguen^10^ and the major concept of bipolar soft sets was invented by Mahmood^11^. The fuzzy soft sets (FSSs) were developed by Ali^12^ and the topological structure of FSS was exposed by Tanay and Kandemir^13^. More, the main idea of interval-valued FS was utilized by Feng et al.^14^. The portfolio investigation based on fuzzy entropy measures was diagnosed by Qin et al.^15^ and two different types of entropy measures for fuzzy data were utilized by Bi et al.^16^.

The desire to address ambiguity and vagueness in decision-making, create workable solutions for genuine issues, and enhance the theoretical underpinnings of this fuzzy set extension motivates researchers in the subject of IFSs. They are working to develop tools that are more reliable and adaptable for dealing with uncertainty in diverse situations. Xu and Yager^17^ devised geometric AOs within intuitionistic fuzzy (IF) information and Xu^18^ deduced AOs under the setting of IF information. The generalized AOs within IFS were devised by Zhao et al.^19^. Xia et al.^20^ deliberated various AOs under IF information by employing Archimedean t-conorm and t-norm. The weighted Maclaurin symmetric mean (MSM) operators within IFS were revealed by Shi and Xiao^21^. Wang et al.^22^ devised an IF PDMSM operator for coping with MADM dilemmas. Liu and Qin^23^ deduced MSM operators in the environment of linguistic IF set. Garg and Arora^24^ devised generalized MSM operators in the environment of the IF soft set. Further, various researchers developed various approaches within IFS such as Ecer^25^ devised the technique of MAIRCA for IFS, Chen, and Tsao^26^ devised the technique of TOPSIS for IFS, Zhao et al.^27^ devised a method of IF MABAC Chatterjee et al.^28^ devised the VIKOR procedure for IFS, and Zhang et al.^29^ interpreted a technique of MULTIMOORA for IFS. The entropy and similarity measures based on IFS were utilized by Thao and Chou^30^ and the histogram equalization based on IFS was presented by Jebadass and Balasubramaniam^31^. The main model for the evaluation of three-way approximation using IFS was anticipated by Yang et al.^32^.

In the area of PFSs, various researchers interpreted new AOs, approaches, and applications to cope with complex vague, and uncertain data in numerous fields. Garg^33^ and Wei^34^ devised various AOs within picture fuzzy (PF) information. Jan et al.^35^ devised Dombi AOs within PF information and Wei^36^ interpreted Hamacher AOs under the setting of PF information. Ullah^37^ devised MSM operators for PF information. Various researchers developed numerous approaches in the environment of PFS like Simic et al.^38^ devised a multi-criteria DM (MCDM) approach for PFS, Jin et al.^39^ devised a technique of TOPSIS for PF information, Meksavang et al.^40^ interpreted an approach of VIKOR for PFS, Liang et al.^41^ deduced method of EDAS-ELECTRE for PF information, Akram et al.^42^ devised COPRAS approach for IFS and Tian et al.^43^ interpreted a technique of MULTIMOORA for IF information.

### Motivation

The theory of MSM was deduced by Maclaurin^44^ and then modified by DeTemple and Roberston^45^. The MSM and dual MSM (DMSM) differ from typical aggregation operators in that they take into account the interactions between many input parameters. Because of this, the MSM and DMSM excel at delivering flexible and reliable data combinations, making it especially useful for dealing with (MADM) scenarios where the characteristics are distinct from one another. Further, to organize and optimize data storage and retrieval, a collection must be partitioned. Combining similar information, cutting down on duplication, and enhancing query efficiency, enables effective data management. Partitioning can also boost parallel processing capacities, resulting in quicker data processing and better system scalability. From the above-mentioned discussion, we observed that there are no AOs under the setting of PF information which is based on DMSM and can cope with the partitioned collection. Thus, in this manuscript, we will try to utilize PDMSM operators in the setting of PF information. The PDMSM operators in the environment of PF information have the supremacy that can justification of the interrelationship of distinct characteristics and there are a lot of exploration consequences for it. Moreover, there is no such MADM approach in the literature under PF information that can employ the PDMSM operator and cope with MADM dilemmas. So further, in this manuscript, we devise a MADM method under PF information relying on the invented PDMSM operators. The basic inspiration for this identification is described below:

1. To develop the novel theory of PFPDMSM operator, WPFPDMSM operator and identified their important results (Idempotency, Monotonicity, Boundedness).
2. To identify the best decision, every expert realized that they needed the best way to find the beneficial optimal using the proper decision-making procedure, for this, we diagnosed the MADM tool in the consideration of deliberated approaches based on PF information.
3. To drive the characteristics of the invented work, several examples are utilized to test the manifest of the comparative analysis with various more existing theories, which is a fascinating and meaningful technique to deeply explain the features and exhibited of the proposed approaches.

### Organization of the paper

Various and main important of this analysis are organized the shape: In Sect. "Preliminaries", we revised the concept of PF data along with their operational laws. Further, we also recalled the theory of Maclaurin symmetric mean (MSM), dual MSM (DMSM), and PDMSM operators and their related results which are very helpful for the invented work. In Sect. "PDMSM Operators Based on PFNs", we established the novel theory of PFPDMSM operator, and WPFPDMSM operator and identified their important results (Idempotency, Monotonicity, Boundedness). In Sect. "Application (“MADM Process”)", we identified the best decision, every expert realized that they needed the best way to find the beneficial optimal using the proper decision-making procedure, for this, we diagnosed the MADM tool in the consideration of deliberated approaches based on PF information. Finally, to drive the characteristics of the invented work, several examples are utilized to test the manifest of the comparative analysis with various more existing theories, which is a fascinating and meaningful technique to deeply explain the features and exhibited of the proposed approaches. In Sect. "Conclusion", we concluded some remarks.

## Preliminaries

The major impact of this scenario is to revise the theory of PF set and its operational rules. Further, we also recalled the theory of Maclaurin symmetric mean (MSM), dual MSM (DMSM), and PDMSM operators and their related results which are very helpful for the invented work.

### Definition 1:

^4^ A PFS $\widehat{\mathcal{M}}$ on $\widetilde{X}$ is deliberated by:

1 $$ \widehat{{\mathcal{M}}} = \left\{ {\langle x,\zeta _{{\mathcal{M}}} \left( x \right),\wp _{{\mathcal{M}}} \left( x \right),\eta _{{\mathcal{M}}} \left( x \right)|x \in \widetilde{X}\rangle } \right\} $$

with $0\le {\zeta }_{\mathcal{M}}\left(x\right)+{\wp }_{\mathcal{M}}\left(x\right)+{\eta }_{\mathcal{M}}\left(x\right)\le 1$. Where ${\zeta }_{\mathcal{M}}\left(x\right),{\wp }_{\mathcal{M}}\left(x\right), {\eta }_{\mathcal{M}}\left(x\right)\in \left[0, 1\right]$ denote the MD, AD and NMD respectively, the triplet $\left({\zeta }_{\mathcal{M}}\left(x\right),{\wp }_{\mathcal{M}}\left(x\right), {\eta }_{\mathcal{M}}\left(x\right)\right)$ is termed PFN. In short, PFN written as $\widehat{\mathcal{M}}=\left(\zeta ,\wp ,\eta \right)$. Further, let ${\widehat{\mathcal{M}}}_{1}=\left({\zeta }_{1}, {\wp }_{1}, {\eta }_{1}\right)$ and ${\widehat{\mathcal{M}}}_{2}=\left({\overline{\zeta }}_{2}, {\overline{\wp } }_{2}, {\overline{\eta }}_{2}\right)$ be two PFNs, $\mathrm{\mho }$ be a positive real number, then

2 $${\widehat{\mathcal{M}}}_{1}\oplus {\widehat{\mathcal{M}}}_{2}=\left(1-\left(1-{\zeta }_{1}\right)\left(1-{\zeta }_{2}\right),{\wp }_{1}{\wp }_{2}, {\eta }_{1}{\eta }_{2}\right)$$

3 $${\widehat{\mathcal{M}}}_{1}\oplus{\widehat{\mathcal{M}}}_{2}=\left({\zeta }_{1}{\zeta }_{2}, 1-\left(1-{\wp }_{1}\right)\left(1-{\wp }_{2}\right), 1-\left(1-{\eta }_{1}\right)\left(1-{\eta }_{2}\right)\right)$$

4 $$\mho {\widehat{\mathcal{M}}}_{1}=\left(1-{\left(1-{\zeta }_{1}\right)}^{\mho },{\wp }_{1}^{\mho }, {\eta }_{1}^{\mho }\right)$$

5 $${\widehat{\mathcal{M}}}_{1}^{\mho }=\left({\zeta }_{1}^{\mho },1-{\left(1-{\wp }_{1}\right)}^{\mho },1-{\left(1-{\eta }_{1}\right)}^{\mho }\right)$$

Moreover, using Eqs. (2)–(5), we obtain the following theories, such that.

(1) $${\widehat{\mathcal{M}}}_{1}\oplus {\widehat{\mathcal{M}}}_{2}={\widehat{\mathcal{M}}}_{2}\oplus {\widehat{\mathcal{M}}}_{1},$$

(2) $${\widehat{\mathcal{M}}}_{1}\otimes {\widehat{\mathcal{M}}}_{2}={\widehat{\mathcal{M}}}_{2}\otimes {\widehat{\mathcal{M}}}_{1},$$

(3) $$\mathrm{\mho }\left({\widehat{\mathcal{M}}}_{1}\oplus {\widehat{\mathcal{M}}}_{2}\right)={\mathrm{\mho }\widehat{\mathcal{M}}}_{1}\oplus {\mathrm{\mho }\widehat{\mathcal{M}}}_{2},\mathrm{ \mho }>0,$$

(4) $${\mathrm{\mho }}_{1}{\widehat{\mathcal{M}}}_{1}\oplus {{\mathrm{\mho }}_{2}\widehat{\mathcal{M}}}_{1}=\left({\mathrm{\mho }}_{1}+{\mathrm{\mho }}_{2}\right){\widehat{\mathcal{M}}}_{1},{\mathrm{\mho }}_{1},{\mathrm{\mho }}_{2}>0,$$

(5) $$ {\widehat{\mathcal{M}}}_{1}^{{\mho_{1} }} \otimes {\widehat{\mathcal{M}}}_{1}^{{\mho_{2} }} = \left( {{\widehat{\mathcal{M}}}_{1} } \right)^{{\mho_{1} + \mho_{2} }} ,{ }\mho_{1} ,\mho_{2} > 0, $$

(6) $$ \left( {\widehat{\mathcal{M}}}_{1} \otimes {\widehat{\mathcal{M}}}_{2} \right)^{\mho } = {\widehat{\mathcal{M}}}_{1}^{\mho } \otimes {\widehat{\mathcal{M}}}_{2}^{\mho } ,{ }\mho > 0 $$

Additionally, finding the ranking between any PFNs is a very challenging task for experts, for this, we revise the score value (SV) and accuracy value (AV), such that

6 $${\mathbb{S}}\left(\widehat{\mathcal{M}}\right)={\zeta }_{\mathcal{M}}\left(x\right)-{\wp }_{\mathcal{M}}\left(x\right){- \eta }_{\mathcal{M}}\left(x\right)\in \left[-1, 1\right]$$

7 $$\check{{\rm A}}\left(\widehat{\mathcal{M}}\right)={\zeta }_{\mathcal{M}}\left(x\right)+{\wp }_{\mathcal{M}}\left(x\right){+ \eta }_{\mathcal{M}}\left(x\right)\in \left[0, 1\right]$$

Then by employing Eqs. (6) and (7), we diagnose.

1. If $${\mathbb{S}}\left({\widehat{\mathcal{M}}}_{1}\right)>{\mathbb{S}}\left({\widehat{\mathcal{M}}}_{2}\right)$$ then $${\widehat{\mathcal{M}}}_{1}>{\widehat{\mathcal{M}}}_{2}$$

2. If $${\mathbb{S}}\left({\widehat{\mathcal{M}}}_{1}\right)={\mathbb{S}}\left({\widehat{\mathcal{M}}}_{2}\right)$$ then.

3. If $$\mathop {\text{A}}\limits^{ \vee } \left( {{\widehat{\mathcal{M}}}_{1} } \right) > \mathop {\text{A}}\limits^{ \vee } \left( {{\widehat{\mathcal{M}}}_{2} } \right)$$ then $${\widehat{\mathcal{M}}}_{1} > {\widehat{\mathcal{M}}}_{2}$$

4. If $$\mathop {\text{A}}\limits^{ \vee } \left( {{\widehat{\mathcal{M}}}_{1} } \right) = \mathop {\text{A}}\limits^{ \vee } \left( {{\widehat{\mathcal{M}}}_{2} } \right)$$ then $${\widehat{\mathcal{M}}}_{1} = {\widehat{\mathcal{M}}}_{2}$$

### Definition 2:

^44^ For any specified positive real numbers ${\mathcal{M}}_{{t}}\left({t}=1, 2,\dots ,{{\mathbbm{f}}}\right),$ and ${{\mathbb{F}}}=1, 2, \ldots .,{{\mathbbm{f}}}$, The MSM operator initiated by:

8 $$MS{M}^{\left({{\mathbb{F}}}\right)}\left({\mathcal{M}}_{1}, {\mathcal{M}}_{2}, \ldots.., {\mathcal{M}}_{{\mathbbm{f}}}\right)={\left(\frac{\sum_{1\le {{t}}_{1}<\cdots <{{t}}_{{\mathbb{F}}}\le {{\mathbbm{f}}}}\left(\prod_{{\mathbb{Y}}=1}^{{\mathbb{F}}}{\mathcal{M}}_{{{t}}_{\mathbb{Y}}}\right)}{{\mathfrak{B}}_{{\mathbbm{f}}}^{{\mathbb{F}}}}\right)}^{\frac{1}{{\mathbb{F}}}}$$

The DMSM operator initiated by:

9 $$DMS{M}^{\left({{\mathbb{F}}}\right)}\left({\mathcal{M}}_{1}, {\mathcal{M}}_{2}, \ldots.., {\mathcal{M}}_{{\mathbbm{f}}}\right)=\frac{1}{{\mathbb{F}}}\left(\prod_{1\le {{t}}_{1}<\cdots <{{t}}_{{\mathbb{F}}}\le {{\mathbbm{f}}}}{\left(\sum_{{\mathbb{Y}}=1}^{{\mathbb{F}}}{\mathcal{M}}_{{{t}}_{\mathbb{Y}}}\right)}^{\frac{1}{{\mathfrak{B}}_{{\mathbbm{f}}}^{{\mathbb{F}}}}}\right)$$

where $\left({{t}}_{1}, {{t}}_{2},\ldots ,{{t}}_{{\mathbbm{F}}}\right)$ covers all the b-tuple combination of $\left(1, 2, . . ,{{\mathbbm{f}}}\right)$ and ${\mathfrak{B}}_{{\mathbbm{f}}}^{{\mathbb{F}}}=\frac{{{\mathbbm{f}}}!}{{{\mathbb{F}}}!\left({{\mathbbm{f}}}-{{\mathbb{F}}}\right)!}$, with various properties, such that.

1. If $${\mathcal{M}}_{{t}}=\mathcal{M}\left({t}=1, 2,\ldots,{{\mathbbm{f}}}\right),$$ then $$DMS{M}^{({{\mathbb{F}}})}\left({\mathcal{M}}_{1}, {\mathcal{M}}_{2}, \ldots, {\mathcal{M}}_{{\mathbbm{f}}}\right)=\mathcal{M},$$
2. If $${\mathcal{M}}_{{t}}\le {\gamma }_{{t}}\left({t}=1, 2,\ldots,{{\mathbbm{f}}}\right)$$, then $$DMS{M}^{({{\mathbb{F}}})}\left({\mathcal{M}}_{1}, {\mathcal{M}}_{2}, \ldots, {\mathcal{M}}_{{\mathbbm{f}}}\right)\le DMS{M}^{({{\mathbb{F}}})}\left({\gamma }_{1}, {\gamma }_{2}, \ldots , {\gamma }_{{\mathbbm{f}}}\right)$$, $$\underset{{t}}{\mathrm{min}}\left\{{\mathcal{M}}_{{t}}\right\}\le DMS{M}^{\left({{\mathbb{F}}}\right)}\left({\mathcal{M}}_{1}, {\mathcal{M}}_{2}, \ldots, {\mathcal{M}}_{{\mathbbm{f}}}\right)\le \underset{{t}}{\mathrm{max}}\left\{{\mathcal{M}}_{{t}}\right\}.$$

### Definition 3:

^22^ The PDMSM operator would be deduced as:

10 $$PDMS{M}^{\left({{\mathbb{F}}}\right)}\left({\mathcal{M}}_{1}, {\mathcal{M}}_{2}, \ldots . ., {\mathcal{M}}_{{\mathbbm{f}}}\right)=\frac{1}{{\acute{c}} }\sum_{{r}=1}^{{\acute{c}} }\left(\frac{\prod_{1\le {{t}}_{1}<\cdots <{{t}}_{{\mathbb{F}}}\le {\varsigma }_{{r}}}{\left(\sum_{{\mathbb{Y}}=1}^{{\mathbb{F}}}{\mathcal{M}}_{{{t}}_{\mathbb{Y}}}\right)}^{\frac{1}{{\mathfrak{B}}_{{\varsigma }_{{r}}}^{{\mathbb{F}}}}}}{{\mathbb{F}}}\right)$$

where $\left({{t}}_{1}, {{t}}_{2},\ldots ,{{t}}_{{\mathbb{F}}}\right)$ holds all the b-tuple combination of $\left(1, 2, . . ,{\varsigma }_{{r}}\right)$ and ${\mathfrak{B}}_{{\varsigma }_{{r}}}^{{\mathbb{F}}}=\frac{{\varsigma }_{{r}}!}{{{\mathbb{F}}}!\left({\varsigma }_{{r}}-{{\mathbb{F}}}\right)!}$, with some results, such that.

### Proposition 1:

^22^ (Idempotency) Let ${\mathcal{M}}_{1}= {\mathcal{M}}_{2}= \cdots={\mathcal{M}}_{{\mathbbm{f}}}=\mathcal{M}.$ Then.

11 $$PDMS{M}^{\left({{\mathbb{F}}}\right)}\left({\mathcal{M}}_{1}, {\mathcal{M}}_{2}, . . . . ., {\mathcal{M}}_{{\mathbbm{f}}}\right)=\mathcal{M}$$

### Proposition 2:

^22^ (Monotonicity) Let ${\chi }_{{t}},{\mathcal{M}}_{{t}}\left({t}=1, 2,\dots ,{{\mathbbm{f}}}\right)$ be two sets of non-negative real numbers and ${\chi }_{{t}}\le {\mathcal{M}}_{{t}},{t}=1, 2,\dots ,{{\mathbbm{f}}},$ then.

12 $$PDMS{M}^{\left({{\mathbb{F}}}\right)}\left({\chi }_{1}, {\chi }_{2}, . . ., {\chi }_{{\mathbbm{f}}}\right)\le PDMS{M}^{\left({{\mathbb{F}}}\right)}\left({\mathcal{M}}_{1}, {\mathcal{M}}_{2}, . . ., {\mathcal{M}}_{{\mathbbm{f}}}\right)$$

when the parameter ${{\mathbb{F}}}$ is fixed.

### Proposition 3:

^22^ (Boundedness) Let ${\mathcal{M}}_{{t}}\left({t}=1, 2,\dots ,{{\mathbbm{f}}}\right)$ be a group of non-negative real numbers, ${\mathcal{M}}^{-}=\underset{{t}}{\mathrm{min}}\left\{{\mathcal{M}}_{{t}}\right\}$ and ${\mathcal{M}}^{+}=\underset{{t}}{\mathrm{max}}\left\{{\mathcal{M}}_{{t}}\right\}$, then.

13 $${\mathcal{M}}^{-}\le PDMS{M}^{\left({{\mathbb{F}}}\right)}\left({\mathcal{M}}_{1}, {\mathcal{M}}_{2}, . . ., {\mathcal{M}}_{{\mathbbm{f}}}\right)\le {\mathcal{M}}^{+}$$

## PDMSM operators based on PFNs

PDMSM operator has the supremacy which can take justification of interrelationship of distinct characteristics and there are a lot of exploration consequences on it. However, it has not been employed to manage MADM dilemmas represented by PFNs. The basic inspiration for this identification is to develop the novel theory of the PFPDMSM operator, and WPFPDMSM operator and identify their important results (Idempotency, Monotonicity, Boundedness).

### Definition 4:

Let a gathering of PFNs ${\widehat{\mathcal{M}}}_{{t}}=\left({\zeta }_{{t}}, {\wp }_{{t}}, {\eta }_{{t}}\right)\left({t}=1, 2,\dots ,{{\mathbbm{f}}}\right)$. Then the underneath expression.

14 $$PFPDMS{M}^{\left({{\mathbb{F}}}\right)}\left({\widehat{\mathcal{M}}}_{1}, {\widehat{\mathcal{M}}}_{2}, \ldots, {\widehat{\mathcal{M}}}_{{\mathbbm{f}}}\right)=\frac{1}{{\acute{c}} }\left(\genfrac{}{}{0pt}{}{{\acute{c}} }{\genfrac{}{}{0pt}{}{\oplus }{{{\mathbb{F}}}=1}}\left(\frac{\genfrac{}{}{0pt}{}{\otimes }{1\le {{t}}_{1}<\cdots <{{t}}_{{\mathbb{F}}}\le {\varsigma }_{{r}}}{\left(\genfrac{}{}{0pt}{}{{\mathbb{F}}}{\genfrac{}{}{0pt}{}{\oplus }{{\mathbb{Y}}=1}}\widehat{\mathcal{M}}_{{{t}}_{\mathbb{Y}}}\right)}^{\frac{1} {{\mathfrak{B}}_{{\varsigma r}}^{{\mathbb{F}}} } }}{{\mathbb{F}}}\right)\right)$$

demonstrate PFPDMSM operators. Where ${\acute{c}}$ reveals number of partitions ${\rm P}\kern-5pt \raisebox{8.5pt}{\rotatebox{180}{J}}{1} , {\rm P}\kern-5pt \raisebox{8.5pt}{\rotatebox{180}{J}}_{2} , \ldots , {\rm P}\kern-5pt \raisebox{8.5pt}{\rotatebox{180}{J}}_{{\acute{c}}}$, ${{\mathbb{F}}}=1, 2, . . . .,$ is a parameter, $\varsigma_{r}$ is the amount of attributes in $P_{r}$,$\left({{t}}_{1}, {{t}}_{2},. . . ,{{t}}_{{\mathbb{F}}}\right)$ covers all the b-tuple combination of $\left(1, 2, . . ,{\varsigma }_{{r}}\right)$ and ${\mathfrak{B}}_{{\varsigma }_{{r}}}^{{\mathbb{F}}}=\frac{{\varsigma }_{{r}}!}{{{\mathbb{F}}}!\left({\varsigma }_{{r}}-{{\mathbb{F}}}\right)!}$.

### Theorem 1:

Let a gathering of PFNs ${\widehat{\mathcal{M}}}_{{t}}=\left({\overline{\zeta }}_{{t}}, {\overline{\wp } }_{{t}}, {\overline{\eta }}_{{t}}\right)\left({t}=1, 2,\dots ,{{\mathbbm{f}}}\right)$. Then by employing Eq. (14), we get.

15 $$PFPDMS{M}^{\left({{\mathbb{F}}}\right)}\left({\widehat{\mathcal{M}}}_{1}, {\widehat{\mathcal{M}}}_{2}, . . ., {\widehat{\mathcal{M}}}_{{\mathbbm{f}}}\right)={\left(\begin{array}{c}1-{\left({\prod_{{r}=1}^{{\acute{c}} }\left(1-\prod_{1\le {{t}}_{1}<. . . <{{t}}_{{\mathbb{F}}}\le {\varsigma }_{{r}}}{\left(1-\prod_{{\mathbb{Y}}=1}^{{\mathbb{F}}}\left(1-{{\zeta }_{{t}}}_{\mathbb{Y}}\right)\right)}^{\frac{1}{{\mathfrak{B}}_{{\varsigma }_{{r}}}^{{\mathbb{F}}}}}\right)}^{\frac{1}{{\mathbb{F}}}}\right)}^{\frac{1}{{\acute{c}} }},\\ {\left({\prod_{{r}=1}^{{\acute{c}} }\left(1-\prod_{1\le {{t}}_{1}<. . . <{{t}}_{{\mathbb{F}}}\le {\varsigma }_{{r}}}{\left(1-\prod_{{\mathbb{Y}}=1}^{{\mathbb{F}}}{{\wp }_{{t}}}_{\mathbb{Y}}\right)}^{\frac{1}{{\mathfrak{B}}_{{\varsigma }_{{r}}}^{{\mathbb{F}}}}}\right)}^{\frac{1}{{\mathbb{F}}}}\right)}^{\frac{1}{{\acute{c}} }}, \\ {\left({\prod_{{r}=1}^{{\acute{c}} }\left(1-\prod_{1\le {{t}}_{1}<. . . <{{t}}_{{\mathbb{F}}}\le {\varsigma }_{{r}}}{\left(1-\prod_{{\mathbb{Y}}=1}^{{\mathbb{F}}}{{\eta }_{{t}}}_{\mathbb{Y}}\right)}^{\frac{1}{{\mathfrak{B}}_{{\varsigma }_{{r}}}^{{\mathbb{F}}}}}\right)}^{\frac{1}{{\mathbb{F}}}}\right)}^{\frac{1}{{\acute{c}} }}\end{array}\right)}$$

### Proof:

Using the operational laws of PFNs, we have.

$$\genfrac{}{}{0pt}{}{{\mathbb{F}}}{\genfrac{}{}{0pt}{}{\oplus}{{\mathbb{Y}}=1}}{\widehat{\mathcal{M}}}_{{{t}}_{\mathbb{Y}}}=\left(1-\prod_{{\mathbb{Y}}=1}^{{\mathbb{F}}}\left(1-{\zeta}_{{{t}}_{\mathbb{Y}}}\right),\prod_{{\mathbb{Y}}=1}^{{\mathbb{F}}}{\wp}_{{{t}}_{\mathbb{Y}}},\prod_{{\mathbb{Y}}=1}^{{\mathbb{F}}}{\eta}_{{{t}}_{\mathbb{Y}}}\right)$$

and

$${\left(\genfrac{}{}{0pt}{}{{\mathbb{F}}}{\genfrac{}{}{0pt}{}{\oplus}{{\mathbb{Y}}=1}}{\widehat{\mathcal{M}}}_{{t}_{\mathbb{Y}}}\right)}^{\frac{1}{{\mathfrak{B}}_{{\varsigma}_{{r}}}^{{\mathbb{F}}}}}=\left({\left(1-\prod_{{\mathbb{Y}}=1}^{{\mathbb{F}}}\left(1-{\zeta}_{{{t}}_{\mathbb{Y}}}\right)\right)}^{\frac{1}{{\mathfrak{B}}_{{\varsigma}_{{r}}}^{{\mathbb{F}}}}},1-{\left(1-\prod_{{\mathbb{Y}}=1}^{{\mathbb{F}}}{\wp}_{{{t}}_{\mathbb{Y}}}\right)}^{\frac{1}{{\mathfrak{B}}_{{\varsigma}_{{r}}}^{{\mathbb{F}}}}},1-{\left(1-\prod_{{\mathbb{Y}}=1}^{{\mathbb{F}}}{\eta}_{{{t}}_{\mathbb{Y}}}\right)}^{\frac{1}{{\mathfrak{B}}_{{\varsigma}_{{r}}}^{{\mathbb{F}}}}}\right)$$

Then we get

$$\genfrac{}{}{0pt}{}{\otimes }{1\le {{t}}_{1}<\cdots<{{t}}_{{\mathbb{F}}}\le {\varsigma}_{{r}}}{\left(\genfrac{}{}{0pt}{}{{\mathbb{F}}}{\genfrac{}{}{0pt}{}{\oplus}{{\mathbb{Y}}=1}}{\widehat{\mathcal{M}}}_{{{t}}_{\mathbb{Y}}}\right)}^{\frac{1}{{\mathfrak{B}}_{{\varsigma}_{{r}}}^{{\mathbb{F}}}}}=\left(\begin{array}{c}\prod_{1\le{{t}}_{1}<\cdots<{{t}}_{{\mathbb{F}}}\le {\varsigma}_{{r}}}{\left(1-\prod_{{\mathbb{Y}}=1}^{{\mathbb{F}}}\left(1-\zeta_{{{t}}_{\mathbb{Y}}}\right)\right)}^{\frac{1}{{\mathfrak{B}}_{{\varsigma}_{{r}}}^{{\mathbb{F}}}}},\\ 1-\prod_{1\le{{t}}_{1}<\cdots<{{t}}_{{\mathbb{F}}}\le {\varsigma}_{{r}}}{\left(1-\prod_{{\mathbb{Y}}=1}^{{\mathbb{F}}}\wp_{{{t}}_{\mathbb{Y}}}\right)}^{\frac{1}{{\mathfrak{B}}_{{\varsigma}_{{r}}}^{{\mathbb{F}}}}},\\ 1-\prod_{1\le{{t}}_{1}<\cdots<{{t}}_{{\mathbb{F}}}\le {\varsigma}_{{r}}}{\left(1-\prod_{{\mathbb{Y}}=1}^{{\mathbb{F}}}\eta_{{{t}}_{\mathbb{Y}}}\right)}^{\frac{1}{{\mathfrak{B}}_{{\varsigma}_{{r}}}^{{\mathbb{F}}}}}\end{array}\right)$$

And

$$\frac{1}{{\mathbb{F}}}\left(\frac{\genfrac{}{}{0pt}{}{\otimes}{1\le {{t}}_{1}<\cdots <{{t}}_{{\mathbb{F}}}\le {\varsigma }_{{r}}}{\left(\genfrac{}{}{0pt}{}{{\mathbb{F}}}{\genfrac{}{}{0pt}{}{\oplus }{{\mathbb{Y}}=1}}\widehat{\mathcal{M}}_{{{t}}_{\mathbb{Y}}}\right)}^{\frac{1}{{\mathfrak{B}}_{{\varsigma}_{{r}}}^{{\mathbb{F}}}}}}{{\mathbb{F}}}\right)=\left(\begin{array}{c}1-{\left(1-\prod_{1\le {{t}}_{1}<\cdots <{{t}}_{{\mathbb{F}}}\le {\varsigma}_{{r}}}{\left(1-\prod_{{\mathbb{Y}}=1}^{{\mathbb{F}}}\left(1- \zeta_{{{t}}_{\mathbb{Y}}}\right)\right)}^{\frac{1}{{\mathfrak{B}}_{{\varsigma }_{{r}}}^{{\mathbb{F}}}}}\right)}^{\frac{1}{{\mathbb{F}}}},\\ {\left(1-\prod_{1\le {{t}}_{1}<\cdots<{{t}}_{{\mathbb{F}}}\le {\varsigma}_{{r}}}{\left(1-\prod_{{\mathbb{Y}}=1}^{{\mathbb{F}}}\wp_{{{t}}_{\mathbb{Y}}}\right)}^{\frac{1}{{\mathfrak{B}}_{{\varsigma}_{{r}}}^{{\mathbb{F}}}}}\right)}^{\frac{1}{{\mathbb{F}}}},\\ {\left(1-\prod_{1\le {{t}}_{1}<\cdots<{{t}}_{{\mathbb{F}}}\le {\varsigma}_{{r}}}{\left(1-\prod_{{\mathbb{Y}}=1}^{{\mathbb{F}}}\eta_{{{t}}_{\mathbb{Y}}}\right)}^{\frac{1}{{\mathfrak{B}}_{{\varsigma}_{{r}}}^{{\mathbb{F}}}}}\right)}^{\frac{1}{{\mathbb{F}}}}\end{array}\right)$$

So, we can get

$$PFPDMS{M}^{\left({{\mathbb{F}}}\right)}\left({\widehat{\mathcal{M}}}_{1}, {\widehat{\mathcal{M}}}_{2}, . . ., {\widehat{\mathcal{M}}}_{{\mathbb{F}}}\right)={\left(\begin{array}{c}1-{\left({\prod_{{r}=1}^{{\acute{c}} }\left(1-\prod_{1\le {{t}}_{1}<. . . <{{t}}_{{\mathbb{F}}}\le {\varsigma }_{{r}}}{\left(1-\prod_{{\mathbb{Y}}=1}^{{\mathbb{F}}}\left(1-{{\zeta }_{{t}}}_{\mathbb{Y}}\right)\right)}^{\frac{1}{{\mathfrak{B}}_{{\varsigma }_{{r}}}^{{\mathbb{F}}}}}\right)}^{\frac{1}{{\mathbb{F}}}}\right)}^{\frac{1}{{\acute{c}} }},\\ {\left({\prod_{{r}=1}^{{\acute{c}} }\left(1-\prod_{1\le {{t}}_{1}<. . . <{{t}}_{{\mathbb{F}}}\le {\varsigma }_{{r}}}{\left(1-\prod_{{\mathbb{Y}}=1}^{{\mathbb{F}}}{{\wp }_{{t}}}_{\mathbb{Y}}\right)}^{\frac{1}{{\mathfrak{B}}_{{\varsigma }_{{r}}}^{{\mathbb{F}}}}}\right)}^{\frac{1}{{\mathbb{F}}}}\right)}^{\frac{1}{{\acute{c}} }}, \\ {\left({\prod_{{r}=1}^{{\acute{c}} }\left(1-\prod_{1\le {{t}}_{1}<. . . <{{t}}_{{\mathbb{F}}}\le {\varsigma }_{{r}}}{\left(1-\prod_{{\mathbb{Y}}=1}^{{\mathbb{F}}}{{\eta }_{{t}}}_{\mathbb{Y}}\right)}^{\frac{1}{{\mathfrak{B}}_{{\varsigma }_{{r}}}^{{\mathbb{F}}}}}\right)}^{\frac{1}{{\mathbb{F}}}}\right)}^{\frac{1}{{\acute{c}} }}\end{array}\right)}$$

Next, we show

$$0\le 1-{\left({\prod_{{r}=1}^{{\acute{c}} }\left(1-\prod_{1\le {{t}}_{1}<. . . <{{t}}_{{\mathbb{F}}}\le {\varsigma }_{{r}}}{\left(1-\prod_{{\mathbb{Y}}=1}^{{\mathbb{F}}}\left(1-{{\zeta }_{{t}}}_{\mathbb{Y}}\right)\right)}^{\frac{1}{{\mathfrak{B}}_{{\varsigma }_{{r}}}^{{\mathbb{F}}}}}\right)}^{\frac{1}{{\mathbb{F}}}}\right)}^{\frac{1}{{\acute{c}} }}\le 1$$

$$0\le {\left({\prod_{{r}=1}^{{\acute{c}} }\left(1-\prod_{1\le {{t}}_{1}<. . . <{{t}}_{{\mathbb{F}}}\le {\varsigma }_{{r}}}{\left(1-\prod_{{\mathbb{Y}}=1}^{{\mathbb{F}}}{{\wp }_{{t}}}_{\mathbb{Y}}\right)}^{\frac{1}{{\mathfrak{B}}_{{\varsigma }_{{r}}}^{{\mathbb{F}}}}}\right)}^{\frac{1}{{\mathbb{F}}}}\right)}^{\frac{1}{{\acute{c}} }}\le 1$$

$${0\le \left({\prod_{{r}=1}^{{\acute{c}} }\left(1-\prod_{1\le {{t}}_{1}<. . . <{{t}}_{{\mathbb{F}}}\le {\varsigma }_{{r}}}{\left(1-\prod_{{\mathbb{Y}}=1}^{{\mathbb{F}}}{{\eta }_{{t}}}_{\mathbb{Y}}\right)}^{\frac{1}{{\mathfrak{B}}_{{\varsigma }_{{r}}}^{{\mathbb{F}}}}}\right)}^{\frac{1}{{\mathbb{F}}}}\right)}^{\frac{1}{{\acute{c}} }}\le 1.$$

To develop AOs that adhere to specific logical and mathematical principles, monotonicity, boundedness, and idempotency serve as a foundation. This improves the usefulness and interpretability of the aggregated data. For aggregation operators to guarantee the dependability and significance of the aggregation process, the properties of monotonicity, boundedness, and idempotency are essential. Monotonicity preserves the intuitive idea that more substantial contributions should result in a bigger aggregate by ensuring that as input values grow, the aggregated output does not decrease. Extreme outliers are prevented from unduly affecting the conclusion by boundedness, which ensures that the aggregate result stays within a certain range. Idempotency guarantees that the same outcome is produced when the AOs are applied again to the same set of data, improving the stability and predictability of the aggregate process. Together, these characteristics provide consistency, precision, and control over the aggregation process, which qualifies it for a variety of applications including statistical analysis, decision-making, and data summarization.

### Proposition 4:

(Idempotency) Let a gathering of PFNs ${\widehat{\mathcal{M}}}_{{t}}=\left({\zeta }_{{t}}, {\wp }_{{t}}, {\eta }_{{t}}\right)\left({t}=1, 2,\dots ,{{\mathbbm{f}}}\right)$. If ${\widehat{\mathcal{M}}}_{{t}}=\left({\zeta }_{{t}}, {\wp }_{{t}}, {\eta }_{{t}}\right)=\widehat{\mathcal{M}}=\left(\zeta , \wp , \eta \right)\left({t}=1, 2,\dots ,{{\mathbbm{f}}}\right),$ then

16 $$PFPDMS{M}^{\left({{\mathbb{F}}}\right)}\left({\widehat{\mathcal{M}}}_{1}, {\widehat{\mathcal{M}}}_{2}, . . ., {\widehat{\mathcal{M}}}_{{\mathbbm{f}}}\right)=\widehat{\mathcal{M}}$$

### Proof:

By using Eq. (15), we have.

$$PFPDMS{M}^{\left({{\mathbb{F}}}\right)}\left({\widehat{\mathcal{M}}}_{1}, {\widehat{\mathcal{M}}}_{2}, . . ., {\widehat{\mathcal{M}}}_{{\mathbbm{f}}}\right)=\left(\begin{array}{c}1-{\left({\prod_{{r}=1}^{{\acute{c}} }\left(1-\prod_{1\le {{t}}_{1}<. . . <{{t}}_{{\mathbb{F}}}\le {\varsigma }_{{r}}}{\left(1-\prod_{{\mathbb{Y}}=1}^{{\mathbb{F}}}\left(1-{{\zeta }_{{t}}}_{\mathbb{Y}}\right)\right)}^{\frac{1}{{\mathfrak{B}}_{{\varsigma }_{{r}}}^{{\mathbb{F}}}}}\right)}^{\frac{1}{{\mathbb{F}}}}\right)}^{\frac{1}{{\acute{c}} }},\\ {\left({\prod_{{r}=1}^{{\acute{c}} }\left(1-\prod_{1\le {{t}}_{1}<. . . <{{t}}_{{\mathbb{F}}}\le {\varsigma }_{{r}}}{\left(1-\prod_{{\mathbb{Y}}=1}^{{\mathbb{F}}}{{\wp }_{{t}}}_{\mathbb{Y}}\right)}^{\frac{1}{{\mathfrak{B}}_{{\varsigma }_{{r}}}^{{\mathbb{F}}}}}\right)}^{\frac{1}{{\mathbb{F}}}}\right)}^{\frac{1}{{\acute{c}} }}, \\ {\left({\prod_{{r}=1}^{{\acute{c}} }\left(1-\prod_{1\le {{t}}_{1}<. . . <{{t}}_{{\mathbb{F}}}\le {\varsigma }_{{r}}}{\left(1-\prod_{{\mathbb{Y}}=1}^{{\mathbb{F}}}{{\eta }_{{t}}}_{\mathbb{Y}}\right)}^{\frac{1}{{\mathfrak{B}}_{{\varsigma }_{{r}}}^{{\mathbb{F}}}}}\right)}^{\frac{1}{{\mathbb{F}}}}\right)}^{\frac{1}{{\acute{c}} }}\end{array}\right)$$

$$=\boldsymbol{ }\boldsymbol{ }\left(\begin{array}{c}1-{\left({\prod_{{r}=1}^{{\acute{c}} }\left(1-\prod_{1\le {{t}}_{1}<. . . <{{t}}_{{\mathbb{F}}}\le {\varsigma }_{{r}}}{\left(1-\prod_{{\mathbb{Y}}=1}^{{\mathbb{F}}}\left(1-\zeta \right)\right)}^{\frac{1}{{\mathfrak{B}}_{{\varsigma }_{{r}}}^{{\mathbb{F}}}}}\right)}^{\frac{1}{{\mathbb{F}}}}\right)}^{\frac{1}{{\acute{c}} }},\\ {\left({\prod_{{r}=1}^{{\acute{c}} }\left(1-\prod_{1\le {{t}}_{1}<. . . <{{t}}_{{\mathbb{F}}}\le {\varsigma }_{{r}}}{\left(1-\prod_{{\mathbb{Y}}=1}^{{\mathbb{F}}}\wp \right)}^{\frac{1}{{\mathfrak{B}}_{{\varsigma }_{{r}}}^{{\mathbb{F}}}}}\right)}^{\frac{1}{{\mathbb{F}}}}\right)}^{\frac{1}{{\acute{c}} }}, \\ {\left({\prod_{{r}=1}^{{\acute{c}} }\left(1-\prod_{1\le {{t}}_{1}<. . . <{{t}}_{{\mathbb{F}}}\le {\varsigma }_{{r}}}{\left(1-\prod_{{\mathbb{Y}}=1}^{{\mathbb{F}}}\eta \right)}^{\frac{1}{{\mathfrak{B}}_{{\varsigma }_{{r}}}^{{\mathbb{F}}}}}\right)}^{\frac{1}{{\mathbb{F}}}}\right)}^{\frac{1}{{\acute{c}} }}\end{array}\right)$$

$$=\boldsymbol{ }\boldsymbol{ }\boldsymbol{ }\left(\begin{array}{c}1-{\left({\prod_{{r}=1}^{{\acute{c}} }\left(1-{\left({\left(1-{\left(1-\zeta \right)}^{{\mathbb{F}}}\right)}^{\frac{1}{{\mathfrak{B}}_{{\varsigma }_{{r}}}^{{\mathbb{F}}}}}\right)}^{{\mathfrak{B}}_{{\varsigma }_{{r}}}^{{\mathbb{F}}}}\right)}^{\frac{1}{{\mathbb{F}}}}\right)}^{\frac{1}{{\acute{c}} }},{\left({\prod_{{r}=1}^{{\acute{c}} }\left(1-{\left({\left({1-\wp }^{{\mathbb{F}}}\right)}^{\frac{1}{{\mathfrak{B}}_{{\varsigma }_{{r}}}^{{\mathbb{F}}}}}\right)}^{{\mathfrak{B}}_{{\varsigma }_{{r}}}^{{\mathbb{F}}}}\right)}^{\frac{1}{{\mathbb{F}}}}\right)}^{\frac{1}{{\acute{c}} }},\\ {\left({\prod_{{r}=1}^{{\acute{c}} }\left(1-{\left({\left({1-\eta }^{{\mathbb{F}}}\right)}^{\frac{1}{{\mathfrak{B}}_{{\varsigma }_{{r}}}^{{\mathbb{F}}}}}\right)}^{{\mathfrak{B}}_{{\varsigma }_{{r}}}^{{\mathbb{F}}}}\right)}^{\frac{1}{{\mathbb{F}}}}\right)}^{\frac{1}{{\acute{c}} }}\end{array}\right)$$

$$=\left(\begin{array}{c}1-{\left({\prod_{{r}=1}^{{\acute{c}} }\left({\left(1-\zeta \right)}^{{\mathbb{F}}}\right)}^{\frac{1}{{\mathbb{F}}}}\right)}^{\frac{1}{{\acute{c}} }},{\left({\prod_{{r}=1}^{{\acute{c}} }\left({\wp }^{{\mathbb{F}}}\right)}^{\frac{1}{{\mathbb{F}}}}\right)}^{\frac{1}{{\acute{c}} }},\\ \end{array}{\left({\prod_{{r}=1}^{{\acute{c}} }\left({\eta }^{{\mathbb{F}}}\right)}^{\frac{1}{{\mathbb{F}}}}\right)}^{\frac{1}{{\acute{c}} }}\right)$$

$$=\left(\zeta , \wp , \eta \right)=\widehat{\mathcal{M}}.$$

### Proposition 5:

(Commutativity) Let a gathering of PFNs ${\widehat{\mathcal{M}}}_{{t}}=\left({\zeta }_{{t}}, {\wp }_{{t}}, {\eta }_{{t}}\right)\left({t}=1, 2,\dots ,{{\mathbb{F}}}\right)$. Then if ${\widehat{\mathcal{M}}}_{{t}}^{\prime}=\left( {\zeta }_{{t}}^{\prime}, {\wp }_{{t}}^{\prime}, {\eta }_{{t}}^{\prime}\right)$ is any permutation of ${\widehat{\mathcal{M}}}_{{t}}=\left({\zeta }_{{t}}, {\wp }_{{t}}, {\eta }_{{t}}\right)\forall {t}$ then,

17 $$PFPDMS{M}^{\left({{\mathbb{F}}}\right)}\left({\widehat{\mathcal{M}}}_{1}, {\widehat{\mathcal{M}}}_{2}, . . ., {\widehat{\mathcal{M}}}_{{\mathbbm{f}}}\right)=PFPDMS{M}^{\left({{\mathbb{F}}}\right)}\left({\widehat{\mathcal{M}}}_{1}^{\prime}, {\widehat{\mathcal{M}}}_{2}^{\prime}, . . ., {\widehat{\mathcal{M}}}_{{\mathbbm{f}}}^{\prime}\right)$$

### Proof:

By using Eq. (15), we get.

$$PFPDMS{M}^{\left({{\mathbb{F}}}\right)}\left({\widehat{\mathcal{M}}}_{1}, {\widehat{\mathcal{M}}}_{2}, . . ., {\widehat{\mathcal{M}}}_{{\mathbbm{f}}}\right)=\left(\begin{array}{c}1-{\left({\prod_{{r}=1}^{\acute{c} }\left(1-\prod_{1\le {{t}}_{1}<. . . <{{t}}_{{\mathbb{F}}}\le {\varsigma }_{{r}}}{\left(1-\prod_{{\mathbb{Y}}=1}^{{\mathbb{F}}}\left(1-{{\zeta }_{{t}}}_{\mathbb{Y}}\right)\right)}^{\frac{1}{{\mathfrak{B}}_{{\varsigma }_{{r}}}^{{\mathbb{F}}}}}\right)}^{\frac{1}{{\mathbb{F}}}}\right)}^{\frac{1}{\acute{c} }},\\ {\left({\prod_{{r}=1}^{\acute{c} }\left(1-\prod_{1\le {{t}}_{1}<. . . <{{t}}_{{\mathbb{F}}}\le {\varsigma }_{{r}}}{\left(1-\prod_{{\mathbb{Y}}=1}^{{\mathbb{F}}}{{\wp }_{{t}}}_{\mathbb{Y}}\right)}^{\frac{1}{{\mathfrak{B}}_{{\varsigma }_{{r}}}^{{\mathbb{F}}}}}\right)}^{\frac{1}{{\mathbb{F}}}}\right)}^{\frac{1}{\acute{c} }}, \\ {\left({\prod_{{r}=1}^{\acute{c} }\left(1-\prod_{1\le {{t}}_{1}<. . . <{{t}}_{{\mathbb{F}}}\le {\varsigma }_{{r}}}{\left(1-\prod_{{\mathbb{Y}}=1}^{{\mathbb{F}}}{{\eta }_{{t}}}_{\mathbb{Y}}\right)}^{\frac{1}{{\mathfrak{B}}_{{\varsigma }_{{r}}}^{{\mathbb{F}}}}}\right)}^{\frac{1}{{\mathbb{F}}}}\right)}^{\frac{1}{\acute{c} }}\end{array}\right)$$

$$PFPDMS{M}^{\left({{\mathbb{F}}}\right)}\left({\widehat{\mathcal{M}}}_{1}^{\prime}, {\widehat{\mathcal{M}}}_{2}^{\prime}, . . ., {\widehat{\mathcal{M}}}_{{\mathbbm{f}}}^{\prime}\right)=\left(\begin{array}{c}1-{\left({\prod_{{r}=1}^{\acute{c} }\left(1-\prod_{1\le {{t}}_{1}<. . . <{{t}}_{{\mathbb{F}}}\le {\varsigma }_{{r}}}{\left(1-\prod_{{\mathbb{Y}}=1}^{{\mathbb{F}}}\left(1-{{\zeta }_{{t}}^{\prime}}_{\mathbb{Y}}\right)\right)}^{\frac{1}{{\mathfrak{B}}_{{\varsigma }_{{r}}}^{{\mathbb{F}}}}}\right)}^{\frac{1}{{\mathbb{F}}}}\right)}^{\frac{1}{\acute{c} }},\\ {\left({\prod_{{r}=1}^{\acute{c} }\left(1-\prod_{1\le {{t}}_{1}<. . . <{{t}}_{{\mathbb{F}}}\le {\varsigma }_{{r}}}{\left(1-\prod_{{\mathbb{Y}}=1}^{{\mathbb{F}}}{{\wp }_{{t}}^{\prime}}_{\mathbb{Y}}\right)}^{\frac{1}{{\mathfrak{B}}_{{\varsigma }_{{r}}}^{{\mathbb{F}}}}}\right)}^{\frac{1}{{\mathbb{F}}}}\right)}^{\frac{1}{\acute{c} }}, \\ {\left({\prod_{{r}=1}^{\acute{c} }\left(1-\prod_{1\le {{t}}_{1}<. . . <{{t}}_{{\mathbb{F}}}\le {\varsigma }_{{r}}}{\left(1-\prod_{{\mathbb{Y}}=1}^{{\mathbb{F}}}{{\eta }_{{t}}^{\prime}}_{\mathbb{Y}}\right)}^{\frac{1}{{\mathfrak{B}}_{{\varsigma }_{{r}}}^{{\mathbb{F}}}}}\right)}^{\frac{1}{{\mathbb{F}}}}\right)}^{\frac{1}{\acute{c} }}\end{array}\right)$$

Because ${\widehat{\mathcal{M}}}_{{t}}^{\prime}=\left({\zeta }_{{t}}^{\prime}, {\wp }_{{t}}^{\prime}, {\eta }_{{t}}^{\prime}\right)$ is any permutation of ${\widehat{\mathcal{M}}}_{{t}}=\left({\zeta }_{{t}}, {\wp }_{{t}}, {\eta }_{{t}}\right)\forall {t}$ So we have $PFPDMS{M}^{\left({{\mathbb{F}}}\right)}\left({\widehat{\mathcal{M}}}_{1}, {\widehat{\mathcal{M}}}_{2}, . . ., {\widehat{\mathcal{M}}}_{{\mathbbm{f}}}\right)=PFPDMS{M}^{\left({{\mathbb{F}}}\right)}\left({\widehat{\mathcal{M}}}_{1}^{\prime}, {\widehat{\mathcal{M}}}_{2}^{\prime}, . . ., {\widehat{\mathcal{M}}}_{{\mathbbm{f}}}^{\prime}\right)$.

### Proposition 6:

(Monotonicity) Let two gatherings of PFNs ${\widehat{\mathcal{M}}}_{{t}}=\left({\zeta }_{{t}}, {\wp }_{{t}}, {\eta }_{{t}}\right)$ and ${\widehat{\mathcal{M}}}_{{t}}^{\prime}=\left({\zeta }_{{t}}^{\prime},{\wp }_{{t}}^{\prime}, {\eta }_{{t}}^{\prime}\right)\left({t}=1, 2,\dots ,{{\mathbbm{f}}}\right)$. If ${\zeta }_{{t}}\ge {\zeta }_{{t}}^{\prime}$,${\wp }_{{t}}\le {\wp }_{{t}}^{\prime}$,${\eta }_{{t}}\le {\eta }_{{t}}^{\prime} \forall {t}$ then,

18 $$PFPDMS{M}^{\left({{\mathbb{F}}}\right)}\left({\widehat{\mathcal{M}}}_{1}, {\widehat{\mathcal{M}}}_{2}, . . ., {\widehat{\mathcal{M}}}_{{\mathbbm{f}}}\right)\ge PFPDMS{M}^{\left({{\mathbb{F}}}\right)}\left({\widehat{\mathcal{M}}}_{1}^{\prime}, {\widehat{\mathcal{M}}}_{2}^{\prime}, . . ., {\widehat{\mathcal{M}}}_{{\mathbbm{f}}}^{\prime}\right)$$

when ${{\mathbb{F}}}$ is fixed.

### Proof:

Let $PFPDMS{M}^{\left({{\mathbb{F}}}\right)}\left({\widehat{\mathcal{M}}}_{1}, {\widehat{\mathcal{M}}}_{2}, . . ., {\widehat{\mathcal{M}}}_{{\mathbbm{f}}}\right)=\widehat{\mathcal{M}}=\left(\zeta , \wp , \eta \right)$ and $PFPDMS{M}^{\left({{\mathbb{F}}}\right)}\left({\widehat{\mathcal{M}}}_{1}^{\prime}, {\widehat{\mathcal{M}}}_{2}^{\prime}, . . ., {\widehat{\mathcal{M}}}_{{\mathbbm{f}}}^{\prime}\right)={\widehat{\mathcal{M}}}^{\prime}=\left({\zeta }^{\prime}, {\wp }^{\prime}, {\eta }^{\prime}\right)$, then.

$$\zeta =1-{\left({\prod_{{r}=1}^{\acute{c} }\left(1-\prod_{1\le {{t}}_{1}<. . . <{{t}}_{{\mathbb{F}}}\le {\varsigma }_{{r}}}{\left(1-\prod_{{\mathbb{Y}}=1}^{{\mathbb{F}}}\left(1-{{\zeta }_{{t}}}_{\mathbb{Y}}\right)\right)}^{\frac{1}{{\mathfrak{B}}_{{\varsigma }_{{r}}}^{{\mathbb{F}}}}}\right)}^{\frac{1}{{\mathbb{F}}}}\right)}^{\frac{1}{\acute{c} }}$$

$${\zeta }^{\prime}=1-{\left({\prod_{{r}=1}^{\acute{c} }\left(1-\prod_{1\le {{t}}_{1}<. . . <{{t}}_{{\mathbb{F}}}\le {\varsigma }_{{r}}}{\left(1-\prod_{{\mathbb{Y}}=1}^{{\mathbb{F}}}\left(1-{{\zeta }_{{t}}^{\prime}}_{\mathbb{Y}}\right)\right)}^{\frac{1}{{\mathfrak{B}}_{{\varsigma }_{{r}}}^{{\mathbb{F}}}}}\right)}^{\frac{1}{{\mathbb{F}}}}\right)}^{\frac{1}{\acute{c} }}$$

$$ \begin{aligned} & \wp ={\left({\prod_{{r}=1}^{\acute{c} }\left(1-\prod_{1\le {{t}}_{1}<. . . <{{t}}_{{\mathbb{F}}}\le {\varsigma }_{{r}}}{\left(1-\prod_{{\mathbb{Y}}=1}^{{\mathbb{F}}}{{\wp }_{{t}}}_{\mathbb{Y}}\right)}^{\frac{1}{{\mathfrak{B}}_{{\varsigma }_{{r}}}^{{\mathbb{F}}}}}\right)}^{\frac{1}{{\mathbb{F}}}}\right)}^{\frac{1}{\acute{c} }},\\ & {\wp }^{\prime}={\left({\prod_{{r}=1}^{\acute{c} }\left(1-\prod_{1\le {{t}}_{1}<. . . <{{t}}_{{\mathbb{F}}}\le {\varsigma }_{{r}}}{\left(1-\prod_{{\mathbb{Y}}=1}^{{\mathbb{F}}}{{\wp }_{{t}}^{\prime}}_{\mathbb{Y}}\right)}^{\frac{1}{{\mathfrak{B}}_{{\varsigma }_{{r}}}^{{\mathbb{F}}}}}\right)}^{\frac{1}{{\mathbb{F}}}}\right)}^{\frac{1}{\acute{c} }}\end{aligned} $$

$$ \begin{aligned} & \eta ={\left({\prod_{{r}=1}^{\acute{c} }\left(1-\prod_{1\le {{t}}_{1}<. . . <{{t}}_{{\mathbb{F}}}\le {\varsigma }_{{r}}}{\left(1-\prod_{{\mathbb{Y}}=1}^{{\mathbb{F}}}{{\eta }_{{t}}}_{\mathbb{Y}}\right)}^{\frac{1}{{\mathfrak{B}}_{{\varsigma }_{{r}}}^{{\mathbb{F}}}}}\right)}^{\frac{1}{{\mathbb{F}}}}\right)}^{\frac{1}{\acute{c} }},\\ & {\eta }^{\prime}={\left({\prod_{{r}=1}^{\acute{c} }\left(1-\prod_{1\le {{t}}_{1}<. . . <{{t}}_{{\mathbb{F}}}\le {\varsigma }_{{r}}}{\left(1-\prod_{{\mathbb{Y}}=1}^{{\mathbb{F}}}{{\eta }_{{t}}^{\prime}}_{\mathbb{Y}}\right)}^{\frac{1}{{\mathfrak{B}}_{{\varsigma }_{{r}}}^{{\mathbb{F}}}}}\right)}^{\frac{1}{{\mathbb{F}}}}\right)}^{\frac{1}{\acute{c} }}\end{aligned} $$

because $${\zeta }_{{t}}\ge {\zeta }_{{t}}^{\prime}$$,$${\wp }_{{t}}\le {\wp }_{{t}}^{\prime}$$,$${\eta }_{{t}}\le {\eta }_{{t}}^{\prime}$$

$$ \begin{aligned}& 1-{\left({\prod_{{r}=1}^{\acute{c} }\left(1-\prod_{1\le {{t}}_{1}<. . . <{{t}}_{{\mathbb{F}}}\le {\varsigma }_{{r}}}{\left(1-\prod_{{\mathbb{Y}}=1}^{{\mathbb{F}}}\left(1-{{\zeta }_{{t}}}_{\mathbb{Y}}\right)\right)}^{\frac{1}{{\mathfrak{B}}_{{\varsigma }_{{r}}}^{{\mathbb{F}}}}}\right)}^{\frac{1}{{\mathbb{F}}}}\right)}^{\frac{1}{\acute{c} }}\\ &\quad\ge 1-{\left({\prod_{{r}=1}^{\acute{c} }\left(1-\prod_{1\le {{t}}_{1}<. . . <{{t}}_{{\mathbb{F}}}\le {\varsigma }_{{r}}}{\left(1-\prod_{{\mathbb{Y}}=1}^{{\mathbb{F}}}\left(1-{{\zeta }_{{t}}^{\prime}}_{\mathbb{Y}}\right)\right)}^{\frac{1}{{\mathfrak{B}}_{{\varsigma }_{{r}}}^{{\mathbb{F}}}}}\right)}^{\frac{1}{{\mathbb{F}}}}\right)}^{\frac{1}{\acute{c} }}\end{aligned} $$

$${\left({\prod_{{r}=1}^{\acute{c} }\left(1-\prod_{1\le {{t}}_{1}<. . . <{{t}}_{{\mathbb{F}}}\le {\varsigma }_{{r}}}{\left(1-\prod_{{\mathbb{Y}}=1}^{{\mathbb{F}}}{{\wp }_{{t}}}_{\mathbb{Y}}\right)}^{\frac{1}{{\mathfrak{B}}_{{\varsigma }_{{r}}}^{{\mathbb{F}}}}}\right)}^{\frac{1}{{\mathbb{F}}}}\right)}^{\frac{1}{\acute{c} }}\le {\left({\prod_{{r}=1}^{\acute{c} }\left(1-\prod_{1\le {{t}}_{1}<. . . <{{t}}_{{\mathbb{F}}}\le {\varsigma }_{{r}}}{\left(1-\prod_{{\mathbb{Y}}=1}^{{\mathbb{F}}}{{\wp }_{{t}}^{\prime}}_{\mathbb{Y}}\right)}^{\frac{1}{{\mathfrak{B}}_{{\varsigma }_{{r}}}^{{\mathbb{F}}}}}\right)}^{\frac{1}{{\mathbb{F}}}}\right)}^{\frac{1}{\acute{c} }}$$

$${\left({\prod_{{r}=1}^{\acute{c} }\left(1-\prod_{1\le {{t}}_{1}<. . . <{{t}}_{{\mathbb{F}}}\le {\varsigma }_{{r}}}{\left(1-\prod_{{\mathbb{Y}}=1}^{{\mathbb{F}}}{{\eta }_{{t}}}_{\mathbb{Y}}\right)}^{\frac{1}{{\mathfrak{B}}_{{\varsigma }_{{r}}}^{{\mathbb{F}}}}}\right)}^{\frac{1}{{\mathbb{F}}}}\right)}^{\frac{1}{\acute{c} }}\le {\left({\prod_{{r}=1}^{\acute{c} }\left(1-\prod_{1\le {{t}}_{1}<. . . <{{t}}_{{\mathbb{F}}}\le {\varsigma }_{{r}}}{\left(1-\prod_{{\mathbb{Y}}=1}^{{\mathbb{F}}}{{\eta }_{{t}}^{\prime}}_{\mathbb{Y}}\right)}^{\frac{1}{{\mathfrak{B}}_{{\varsigma }_{{r}}}^{{\mathbb{F}}}}}\right)}^{\frac{1}{{\mathbb{F}}}}\right)}^{\frac{1}{\acute{c} }}$$

which shows ${\zeta }_{{t}}\ge {\zeta }_{{t}}^{\prime}$,${\wp }_{{t}}\le {\wp }_{{t}}^{\prime}$ and ${\eta }_{{t}}\le {\eta }_{{t}}^{\prime}$ , then $\widehat{\mathcal{M}}\ge {\widehat{\mathcal{M}}}^{\prime}$. So

$$PFPDMS{M}^{\left({{\mathbb{F}}}\right)}\left({\widehat{\mathcal{M}}}_{1}, {\widehat{\mathcal{M}}}_{2}, . . ., {\widehat{\mathcal{M}}}_{{\mathbbm{f}}}\right)\ge PFPDMS{M}^{\left({{\mathbb{F}}}\right)}\left({\widehat{\mathcal{M}}}_{1}^{\prime}, {\widehat{\mathcal{M}}}_{2}^{\prime}, . . ., {\widehat{\mathcal{M}}}_{{\mathbbm{f}}}^{\prime}\right)$$

### Proposition 7:

(Boundedness) Let a gathering of PFNs ${\widehat{\mathcal{M}}}_{{t}}=\left({\zeta }_{{t}}, {\wp }_{{t}}, {\eta }_{{t}}\right)\left({t}=1, 2,\dots ,{{\mathbb{F}}}\right)$. If ${\widehat{\mathcal{M}}}^{-}=\underset{{t}}{\mathrm{min}}\left\{{\widehat{\mathcal{M}}}_{{t}}\right\}$ and ${\widehat{\mathcal{M}}}^{+}=\underset{{t}}{\mathrm{max}}\left\{{\widehat{\mathcal{M}}}_{{t}}\right\},{t}=1, 2,\dots ,{{\mathbbm{f}}}$ then

19 $${\widehat{\mathcal{M}}}^{-}\le PFPDMS{M}^{\left({{\mathbb{F}}}\right)}\left({\widehat{\mathcal{M}}}_{1}, {\widehat{\mathcal{M}}}_{2}, . . ., {\widehat{\mathcal{M}}}_{{\mathbb{F}}}\right)\le {\widehat{\mathcal{M}}}^{+}$$

### Proof:

Owing to ${\widehat{\mathcal{M}}}^{-}=\underset{{t}}{\mathrm{min}}\left\{{\widehat{\mathcal{M}}}_{{t}}\right\}\le {\widehat{\mathcal{M}}}_{{t}}$, also by propositions 4 and 6, we have,

$${\widehat{\mathcal{M}}}^{-}=PFPDMS{M}^{\left({{\mathbb{F}}}\right)}\left({\widehat{\mathcal{M}}}^{-}, {\widehat{\mathcal{M}}}^{-}, . . ., {\widehat{\mathcal{M}}}^{-}\right)\le PFPDMS{M}^{\left({{\mathbb{F}}}\right)}\left({\widehat{\mathcal{M}}}_{1}, {\widehat{\mathcal{M}}}_{2}, . . ., {\widehat{\mathcal{M}}}_{{\mathbbm{f}}}\right)$$

and

$$PFPDMS{M}^{\left({{\mathbb{F}}}\right)}\left({\widehat{\mathcal{M}}}_{1}, {\widehat{\mathcal{M}}}_{2}, . . ., {\widehat{\mathcal{M}}}_{{\mathbbm{f}}}\right)\le PFPDMS{M}^{\left({{\mathbb{F}}}\right)}\left({\widehat{\mathcal{M}}}^{+}, {\widehat{\mathcal{M}}}^{+}, . . ., {\widehat{\mathcal{M}}}^{+}\right)={\widehat{\mathcal{M}}}^{+}$$

So, we have.

$${\widehat{\mathcal{M}}}^{-}\le PFPDMS{M}^{\left({{\mathbb{F}}}\right)}\left({\widehat{\mathcal{M}}}_{1}, {\widehat{\mathcal{M}}}_{2}, . . ., {\widehat{\mathcal{M}}}_{{\mathbbm{f}}}\right)\le {\widehat{\mathcal{M}}}^{+}.$$

Let $${\widehat{\mathcal{M}}}_{{t}}=\left({\zeta }_{{t}}, {\wp }_{{t}}, {\eta }_{{t}}\right)\left({t}=1, 2,\dots ,{{\mathbbm{f}}}\right)$$ be PFNs partitioned into $$\acute{c} $$ distinct partitions $${\rm P}\kern-5pt \raisebox{8.5pt}{\rotatebox{180}{J}}{1} , {\rm P}\kern-5pt \raisebox{8.5pt}{\rotatebox{180}{J}}_{2} , \ldots , {\rm P}\kern-5pt \raisebox{8.5pt}{\rotatebox{180}{J}}_{\acute{c}}$$ weight of PFNs $${\widehat{\mathcal{M}}}_{{t}}\left({t}=1, 2,\dots ,{{\mathbbm{f}}}\right)$$ is $$\raisebox{2.1pt}{$\upomega$}\kern-2.3pt\rotatebox{80}{\tiny $\smile$}_{{t}}$$, holds $$\raisebox{2.1pt}{$\upomega$}\kern-2.3pt\rotatebox{80}{\tiny $\smile$}_{{t}}\in \left[0, 1\right]$$ and $$\sum_{{t}=1}^{{\mathbb{F}}}\raisebox{2.1pt}{$\upomega$}\kern-2.3pt\rotatebox{80}{\tiny $\smile$}_{{t}}=1, {{\mathbb{F}}}=1, 2, . . . .,{\varsigma }_{{r}}, {\varsigma }_{{r}}$$ is the amount of attributes in $${\rm P}\kern-5pt \raisebox{8.5pt}{\rotatebox{180}{J}}_{{r}}.$$

### Definition 5:

Let a gathering of PFNs ${\widehat{\mathcal{M}}}_{{t}}=\left({\zeta }_{{t}}, {\wp }_{{t}}, {\eta }_{{t}}\right)\left({t}=1, 2,\dots ,{{\mathbbm{f}}}\right)$. Then the underneath expression.

20 $$WPFPDMS{M}^{\left({{\mathbb{F}}}\right)}\left({\widehat{\mathcal{M}}}_{1},{\widehat{\mathcal{M}}}_{2}, \cdots,{\widehat{\mathcal{M}}}_{{\mathbbm{f}}}\right)=\frac{1}{\acute{c}}\left(\genfrac{}{}{0pt}{}{\acute{c} }{\genfrac{}{}{0pt}{}{\oplus}{{{\mathbb{F}}}=1}}\left(\frac{\genfrac{}{}{0pt}{}{\otimes }{1\le{{t}}_{1}<\cdots <{{t}}_{{\mathbb{F}}}\le{\varsigma}_{{r}}}{\left(\genfrac{}{}{0pt}{}{{\mathbb{F}}}{\genfrac{}{}{0pt}{}{\oplus}{{\mathbb{Y}}=1}}\left(\raisebox{2.1pt}{$\upomega$}\kern-2.3pt\rotatebox{80}{\tiny$\smile$}_{{{t}}_{\mathbb{Y}}}\widehat{\mathcal{M}}_{{{t}}_{\mathbb{Y}}}\right)\right)}^{\frac{1}{{\mathfrak{B}}_{{\varsigma}_{{r}}}^{{\mathbb{F}}}}}}{{\mathbb{F}}}\right)\right)$$

demonstrate WPFPDMSM operators. Where $\acute{c}$ reveals the number of partitions ${\rm P}\kern-5pt \raisebox{8.5pt}{\rotatebox{180}{J}}_{1},{\rm P}\kern-5pt \raisebox{8.5pt}{\rotatebox{180}{J}}_{2}, . . .,{\rm P}\kern-5pt \raisebox{8.5pt}{\rotatebox{180}{J}}_{\acute{c} }$, ${{\mathbb{F}}}=1, 2, . . . .,{\varsigma }_{{r}}$ is a parameter, ${\varsigma }_{{r}}$ is the amount of attributes in ${\rm P}\kern-5pt \raisebox{8.5pt}{\rotatebox{180}{J}}_{{r}}$,$\left({{t}}_{1}, {{t}}_{2},. . . ,{{t}}_{{\mathbb{F}}}\right)$ contains all the b-tuple combinations of $\left(1, 2, . . ,{\varsigma }_{{r}}\right)$ and ${\mathfrak{B}}_{{\varsigma }_{{r}}}^{{\mathbb{F}}}=\frac{{\varsigma }_{{r}}!}{{{\mathbb{F}}}!\left({\varsigma }_{{r}}-{{\mathbb{F}}}\right)!}$.

### Theorem 2:

Let a gathering of PFNs ${\widehat{\mathcal{M}}}_{{t}}=\left({\overline{\zeta }}_{{t}}, {\overline{\wp } }_{{t}}, {\overline{\eta }}_{{t}}\right)\left({t}=1, 2,\dots ,{{\mathbbm{f}}}\right)$. Then by employing Eq. (20), we get.

21 $$ \begin{aligned} & WPFPDMS{M}^{\left({{\mathbb{F}}}\right)}\left({\widehat{\mathcal{M}}}_{1}, {\widehat{\mathcal{M}}}_{2}, . . ., {\widehat{\mathcal{M}}}_{{\mathbb{F}}}\right)\\ &\quad={\left(\begin{array}{c}1-{\left({\prod_{{r}=1}^{\acute{c} }\left(1-\prod_{1\le {{t}}_{1}<. . . <{{t}}_{{\mathbb{F}}}\le {\varsigma }_{{r}}}{\left(1-\prod_{{\mathbb{Y}}=1}^{{\mathbb{F}}}{\left(1-{{\zeta }_{{t}}}_{\mathbb{Y}}\right)}^{\raisebox{2.1pt}{$\upomega$}\kern-2.3pt\rotatebox{80}{\tiny $\smile$}_{{{t}}_{\mathbb{Y}}}}\right)}^{\frac{1}{{\mathfrak{B}}_{{\varsigma }_{{r}}}^{{\mathbb{F}}}}}\right)}^{\frac{1}{{\mathbb{F}}}}\right)}^{\frac{1}{\acute{c} }},\\ {\left({\prod_{{r}=1}^{\acute{c} }\left(1-\prod_{1\le {{t}}_{1}<. . . <{{t}}_{{\mathbb{F}}}\le {\varsigma }_{{r}}}{\left(1-\prod_{{\mathbb{Y}}=1}^{{\mathbb{F}}}{{\wp }_{{t}}}_{\mathbb{Y}}^{\raisebox{2.1pt}{$\upomega$}\kern-2.3pt\rotatebox{80}{\tiny $\smile$}_{{{t}}_{\mathbb{Y}}}}\right)}^{\frac{1}{{\mathfrak{B}}_{{\varsigma }_{{r}}}^{{\mathbb{F}}}}}\right)}^{\frac{1}{{\mathbb{F}}}}\right)}^{\frac{1}{\acute{c} }}, \\ {\left({\prod_{{r}=1}^{\acute{c} }\left(1-\prod_{1\le {{t}}_{1}<. . . <{{t}}_{{\mathbb{F}}}\le {\varsigma }_{{r}}}{\left(1-\prod_{{\mathbb{Y}}=1}^{{\mathbb{F}}}{{\eta }_{{t}}}_{\mathbb{Y}}^{\raisebox{2.1pt}{$\upomega$}\kern-2.3pt\rotatebox{80}{\tiny $\smile$}_{{{t}}_{\mathbb{Y}}}}\right)}^{\frac{1}{{\mathfrak{B}}_{{\varsigma }_{{r}}}^{{\mathbb{F}}}}}\right)}^{\frac{1}{{\mathbb{F}}}}\right)}^{\frac{1}{\acute{c} }}\end{array}\right)}\end{aligned} $$

### Proof:

Omitted.

Additionally, we diagnose some properties, such that.

### Proposition 8:

(Commutativity) Let a gathering of PFNs ${\widehat{\mathcal{M}}}_{{t}}=\left({\zeta }_{{t}}, {\wp }_{{t}}, {\eta }_{{t}}\right)\left({t}=1, 2,\dots ,{{\mathbbm{f}}}\right)$. If ${\widehat{\mathcal{M}}}_{{t}}^{\prime}=\left({\zeta }_{{t}}^{\prime}, {\wp }_{{t}}^{\prime}, {\eta }_{{t}}^{\prime}\right)$ is any permutation of ${\widehat{\mathcal{M}}}_{{t}}=\left({\zeta }_{{t}}, {\wp }_{{t}}, {\eta }_{{t}}\right)\forall {t}$, then

22 $$WPFPDMS{M}^{\left({{\mathbb{F}}}\right)}\left({\widehat{\mathcal{M}}}_{1}, {\widehat{\mathcal{M}}}_{2}, . . ., {\widehat{\mathcal{M}}}_{{\mathbbm{f}}}\right)=WPFPDMS{M}^{\left({{\mathbb{F}}}\right)}\left({\widehat{\mathcal{M}}}_{1}^{\prime}, {\widehat{\mathcal{M}}}_{2}^{\prime}, . . ., {\widehat{\mathcal{M}}}_{{\mathbbm{f}}}^{\prime}\right)$$

### Proposition 9:

(Monotonicity) Let two gatherings of PFNs ${\widehat{\mathcal{M}}}_{{t}}=\left({\zeta }_{{t}}, {\wp }_{{t}}, {\eta }_{{t}}\right)$ and ${\widehat{\mathcal{M}}}_{{t}}^{\prime}=\left({\zeta }_{{t}}^{\prime},{\wp }_{{t}}^{\prime}, {\eta }_{{t}}^{\prime}\right)\left({t}=1, 2,\dots ,{{\mathbbm{f}}}\right)$. If ${\zeta }_{{t}}\ge {\zeta }_{{t}}^{\prime}$, ${\wp }_{{t}}\le {\wp }_{{t}}^{\prime}$, ${\eta }_{{t}}\le {\eta }_{{t}}^{\prime} \forall {t}$ then,

23 $$WPFPDMS{M}^{\left({{\mathbb{F}}}\right)}\left({\widehat{\mathcal{M}}}_{1}, {\widehat{\mathcal{M}}}_{2}, . . ., {\widehat{\mathcal{M}}}_{{\mathbbm{f}}}\right)\ge WPFPDMS{M}^{\left({{\mathbb{F}}}\right)}\left({\widehat{\mathcal{M}}}_{1}^{\prime}, {\widehat{\mathcal{M}}}_{2}^{\prime}, . . ., {\widehat{\mathcal{M}}}_{{\mathbbm{f}}}^{\prime}\right)$$

when ${{\mathbb{F}}}$ is fixed. Further, if we choose the value of $\acute{c} =1,$ then we get

$$ \begin{aligned} & WPFPDMS{M}^{\left(1\right)}\left({\widehat{\mathcal{M}}}_{1}, {\widehat{\mathcal{M}}}_{2}, . . ., {\widehat{\mathcal{M}}}_{{\mathbbm{f}}}\right)\\ &\quad={\left(\begin{array}{c}1-{\left(1-\prod_{1\le {{t}}_{1}<. . . <{{t}}_{{\mathbb{F}}}\le {\varsigma }_{{r}}}{\left(1-\prod_{{\mathbb{Y}}=1}^{{\mathbb{F}}}{\left(1-{{\zeta }_{{t}}}_{\mathbb{Y}}\right)}^{\raisebox{2.1pt}{$\upomega$}\kern-2.3pt\rotatebox{80}{\tiny $\smile$}_{{{t}}_{\mathbb{Y}}}}\right)}^{\frac{1}{{\mathfrak{B}}_{{\varsigma }_{{r}}}^{{\mathbb{F}}}}}\right)}^{\frac{1}{{\mathbb{F}}}},\\ {\left(1-\prod_{1\le {{t}}_{1}<. . . <{{t}}_{{\mathbb{F}}}\le {\varsigma }_{{r}}}{\left(1-\prod_{{\mathbb{Y}}=1}^{{\mathbb{F}}}{{\wp }_{{t}}}_{\mathbb{Y}}^{\raisebox{2.1pt}{$\upomega$}\kern-2.3pt\rotatebox{80}{\tiny $\smile$}_{{{t}}_{\mathbb{Y}}}}\right)}^{\frac{1}{{\mathfrak{B}}_{{\varsigma }_{{r}}}^{{\mathbb{F}}}}}\right)}^{\frac{1}{{\mathbb{F}}}},\\ {\left(1-\prod_{1\le {{t}}_{1}<. . . <{{t}}_{{\mathbb{F}}}\le {\varsigma }_{{r}}}{\left(1-\prod_{{\mathbb{Y}}=1}^{{\mathbb{F}}}{{\eta }_{{t}}}_{\mathbb{Y}}^{\raisebox{2.1pt}{$\upomega$}\kern-2.3pt\rotatebox{80}{\tiny $\smile$}_{{{t}}_{\mathbb{Y}}}}\right)}^{\frac{1}{{\mathfrak{B}}_{{\varsigma }_{{r}}}^{{\mathbb{F}}}}}\right)}^{\frac{1}{{\mathbb{F}}}}\end{array}\right)}\end{aligned} $$

## Application (“MADM process”)

MADM is a crucial and essential part of the decision-making sciences whose aim is to recognize the massive dominant decision from the group of expected ones. In a genuine decision-making strategy, the problem needs to resolve the provided decisions by many classes like single or interval evaluation investigations. However, in many awkward scenarios, it is very challenging for the expert to reduce their decisions to a classical number. However, it has not been employed to manage MADM dilemmas represented by PFNs. The basic inspiration for this identification was to develop the novel theory of PFPDMSM operator, and WPFPDMSM operator and identify their important results (Idempotency, Monotonicity, Boundedness).

The primary goal of this section is to identify the best decision, every expert realized that they needed the best way to find the beneficial optimal using the proper DM procedure, for this, we diagnosed the MADM tool in the consideration of deliberated approaches based on PF information. Finally, to drive the characteristics of the invented work, several examples are utilized to test the manifest of the comparative analysis with numerous more prevailing theories, which is a fascinating and meaningful technique to deeply explain the features and exhibited of the proposed approaches.

### Decision-making technique

In this scenario, we aim to diagnose a new MADM process based on the environment for solving DM dilemmas. For this, we assume $$\mathfrak{T}=\left\{{\mathfrak{T}}_{1}, {\mathfrak{T}}_{2},. . . , {\mathfrak{T}}_{ \underline{{\text{n}}}  }\right\}$$, and $$\Xi =\left\{{\Xi }_{1},{\Xi }_{2},. .. ,{\Xi }_{{\mathbb{F}}}\right\}$$ be a group of alternatives and attributes with $$\raisebox{2.1pt}{$\upomega$}\kern-2.3pt\rotatebox{80}{\tiny $\smile$}=\left\{\raisebox{2.1pt}{$\upomega$}\kern-2.3pt\rotatebox{80}{\tiny $\smile$}_{1},\raisebox{2.1pt}{$\upomega$}\kern-2.3pt\rotatebox{80}{\tiny $\smile$}_{2},. . .,\raisebox{2.1pt}{$\upomega$}\kern-2.3pt\rotatebox{80}{\tiny $\smile$}_{{\mathbbm{f}}}\right\}$$, representing the weight vectors such that $$\sum_{{t}=1}^{{\mathbb{F}}}\raisebox{2.1pt}{$\upomega$}\kern-2.3pt\rotatebox{80}{\tiny $\smile$}_{{t}}=1, {{\mathbb{F}}}=1, 2, . . ., {{\mathbbm{f}}}$$. Using PFNs DMs evaluate the $${\mathfrak{T}}_{{t}}$$ concerning the attribute $${\Xi }_{\mathbb{Y}}$$ and represent it as $${\widehat{\mathcal{M}}}_{{{t}}_{\mathbb{Y}}}=\left({\zeta }_{{{t}}_{\mathbb{Y}}}, {\wp }_{{{t}}_{\mathbb{Y}}}, {\eta }_{{{t}}_{\mathbb{Y}}}\right)$$ where $${\zeta }_{{{t}}_{\mathbb{Y}}}, {\wp }_{{{t}}_{\mathbb{Y}}}, {\eta }_{{{t}}_{\mathbb{Y}}}\in \left[0, 1\right]$$ and $$0\le {\zeta }_{{{t}}_{\mathbb{Y}}}+{\wp }_{{{t}}_{\mathbb{Y}}}+ {\eta }_{{{t}}_{\mathbb{Y}}}\le 1$$. We can acquire a decision matrix $$\mathcal{Y}={\left[{\widehat{\mathcal{M}}}_{{{t}}_{\mathbb{Y}}}\right]}_{ \underline{{\text{n}}}  \times {{\mathbbm{f}}}}$$ ,$$1\le {t}\le  \underline{{\text{n}}}  , 1\le {\mathbbm{y}}\le {{\mathbbm{f}}}$$. Let $$\mathfrak{T}=\left\{{\mathfrak{T}}_{1}, {\mathfrak{T}}_{2},. . . , {\mathfrak{T}}_{ \underline{{\text{n}}}  }\right\}$$ is partitioned into $$\acute{c} $$ distinct groups $${\rm P}\kern-5pt \raisebox{8.5pt}{\rotatebox{180}{J}}_{1}, {\rm P}\kern-5pt \raisebox{8.5pt}{\rotatebox{180}{J}}_{2}, . . .,{\rm P}\kern-5pt \raisebox{8.5pt}{\rotatebox{180}{J}}_{\acute{c} }$$. Therefore, to identify the best decision, every expert realized that they needed the best way to find the beneficial optimal using the proper decision-making procedure. For this, we computed the procedure, whose main steps are defined below.

*Step 1* In DM dilemmas there are two various sort of attributes, that is cost sort and benefit sort, to abolish the effect of different attribute types, transform the decision matrix $\mathcal{Y}={\left[{\widehat{\mathcal{M}}}_{{{t}}_{\mathbb{Y}}}\right]}_{ \underline{{\text{n}}}  \times {{\mathbbm{f}}}}$ into ${\mathcal{Y}}^{\prime}={\left[{\widehat{\mathcal{M}}}_{{{t}}_{\mathbb{Y}}}^{\prime}\right]}_{ \underline{{\text{n}}}  \times {{\mathbbm{f}}}},1\le {t}\le  \underline{{\text{n}}}  , 1\le {\mathbbm{y}}\le {{\mathbbm{f}}}$. To normalize the decision matrix use the given formula:

$${\widehat{\mathcal{M}}}_{{{t}}_{\mathbb{Y}}}^{\prime}=\left({\zeta }_{{{t}}_{\mathbb{Y}}}^{\prime}, {\wp }_{{{t}}_{\mathbb{Y}}}^{\prime}, {\eta }_{{{t}}_{\mathbb{Y}}}^{\prime}\right)=\left\{\begin{array}{c}\left({\zeta }_{{{t}}_{\mathbb{Y}}}, {\wp }_{{{t}}_{\mathbb{Y}}}, {\eta }_{{{t}}_{\mathbb{Y}}}\right), \;  for \;  benefit  \; attribute\\ \left({\eta }_{{{t}}_{\mathbb{Y}}}, {\wp }_{{{t}}_{\mathbb{Y}}}, {\zeta }_{{{t}}_{\mathbb{Y}}}\right),\;   for \;  the \;  cost  \; attribute\end{array}\right.$$

where $$1\le {t}\le  \underline{{\text{n}}}  , 1\le {\mathbbm{y}}\le {{\mathbbm{f}}}$$.

*Step 2* Using the developed WPFPDMSM operator to get

$${\widehat{\mathcal{M}}}_{{t}}^{\prime}=\left({\zeta }_{{t}}^{\prime}, {\wp }_{{t}}^{\prime}, {\eta }_{{t}}^{\prime}\right)=WPFPDMS{M}^{\left({{\mathbb{F}}}\right)}\left({\widehat{\mathcal{M}}}_{1}^{\prime}, {\widehat{\mathcal{M}}}_{2}^{\prime}, . . ., {\widehat{\mathcal{M}}}_{{\mathbbm{f}}}^{\prime}\right)$$

Noted that ${\widehat{\mathcal{M}}}_{{t}}^{\prime}$ is the preference argument of alternative ${\mathfrak{T}}_{{t}}$, ${t}=1, 2,. . ., \underline{{\text{n}}}$

*Step 3* Obtained values ${\mathbb{S}}\left({\widehat{\mathcal{M}}}_{{t}}^{\prime}\right)$ and $\check{{\rm A}}\left({\widehat{\mathcal{M}}}_{{t}}^{\prime}\right)$ of aggregated outcomes ${\widehat{\mathcal{M}}}_{{t}}^{\prime}\left({t}=1, 2,. . ., \underline{{\text{n}}}  \right).$

*Step 4* Make a comparison between ${\mathbb{S}}\left({\widehat{\mathcal{M}}}_{1}^{\prime}\right), {\mathbb{S}}\left({\widehat{\mathcal{M}}}_{2}^{\prime}\right),. . . .,{\mathbb{S}}\left({\widehat{\mathcal{M}}}_{ \underline{{\text{n}}}  }^{\prime}\right)$ and $\check{{\rm A}}\left({\widehat{\mathcal{M}}}_{1}^{\prime}\right), \check{{\rm A}}\left({\widehat{\mathcal{M}}}_{2}^{\prime}\right),. . .,\check{{\rm A}}\left({\widehat{\mathcal{M}}}_{ \underline{{\text{n}}}  }^{\prime}\right)$, then ranking all the alternatives to get the finest one.

### Numerical example

Assume the five suitable enterprise resource management (ERP) schemes expressed by $\left\{{\mathfrak{T}}_{1}, {\mathfrak{T}}_{2},{\mathfrak{T}}_{3},{\mathfrak{T}}_{4},{\mathfrak{T}}_{5}\right\}$ denoted the collection alternatives, which require to be resolved by decision-makers. For this, ERP schemes are resolved based on the four attributes expressed by $\left\{{\Xi }_{1},{\Xi }_{2},{\Xi }_{3},{\Xi }_{4}\right\}$, represented by:${\Xi }_{1}$: Technical Achievement;${\Xi }_{2}$: Human Recourse; ${\Xi }_{3}$: Economic benefits and ${\Xi }_{4}$: constructions of the enterprises. For every attribute, the expert gives their opinion in the shape of a weight vector like 0.4, 0.3, 0.2, and 0.1. Therefore, the selection process of the ERP scheme is evaluated with the help ofthe above procedure.

*Step 1* In DM issues there are two various sort of attributes, that is cost sort and benefit sort,, to abolish the effect of different attribute types, transform the decision matrix $\mathcal{Y}={\left[{\widehat{\mathcal{M}}}_{{{t}}_{\mathbb{Y}}}\right]}_{ \underline{{\text{n}}}  \times {{\mathbbm{f}}}}$ into ${\mathcal{Y}}^{\prime}={\left[{\widehat{\mathcal{M}}}_{{{t}}_{\mathbb{Y}}}^{\prime}\right]}_{ \underline{{\text{n}}}  \times {{\mathbbm{f}}}},1\le {t}\le  \underline{{\text{n}}}  , 1\le {\mathbb{Y}}\le {{\mathbbm{f}}}$, explained in Table 1.

**Table 1:** The decision matrix contains the picture fuzzy information.

	${{\varvec{\Xi}}}_{1}$	${{\varvec{\Xi}}}_{2}$	${{\varvec{\Xi}}}_{3}$	${{\varvec{\Xi}}}_{4}$
${\mathfrak{T}}_{1}$	$\left(\mathrm{0.4,0.2,0.1}\right)$	$\left(\mathrm{0.41,0.21,0.11}\right)$	$\left(\mathrm{0.42,0.22,0.12}\right)$	$\left(\mathrm{0.43,0.23,0.13}\right)$
${\mathfrak{T}}_{2}$	$\left(\mathrm{0.3,0.3,0.2}\right)$	$\left(\mathrm{0.31,0.31,0.21}\right)$	$\left(\mathrm{0.32,0.32,0.22}\right)$	$\left(\mathrm{0.33,0.33,0.23}\right)$
${\mathfrak{T}}_{3}$	$\left(\mathrm{0.5,0.1,0.1}\right)$	$\left(\mathrm{0.51,0.11,0.11}\right)$	$\left(\mathrm{0.52,0.12,0.12}\right)$	$\left(\mathrm{0.53,0.13,0.13}\right)$
${\mathfrak{T}}_{4}$	$\left(\mathrm{0.7,0.01,0.1}\right)$	$\left(\mathrm{0.71,0.02,0.11}\right)$	$\left(\mathrm{0.72,0.03,0.12}\right)$	$\left(\mathrm{0.73,0.04,0.13}\right)$
${\mathfrak{T}}_{5}$	$\left(\mathrm{0.4,0.3,0.1}\right)$	$\left(\mathrm{0.41,0.31,0.11}\right)$	$\left(\mathrm{0.42,0.32,0.12}\right)$	$\left(\mathrm{0.43,0.33,0.13}\right)$

To normalize the decision matrix use the given formula:

$${\widehat{\mathcal{M}}}_{{{t}}_{\mathbb{Y}}}^{\prime}=\left({\zeta }_{{{t}}_{\mathbb{Y}}}^{\prime}, {\wp }_{{{t}}_{\mathbb{Y}}}^{\prime}, {\eta }_{{{t}}_{\mathbb{Y}}}^{\prime}\right)=\left\{\begin{array}{c}\left({\zeta }_{{{t}}_{\mathbb{Y}}}, {\wp }_{{{t}}_{\mathbb{Y}}}, {\eta }_{{{t}}_{\mathbb{Y}}}\right), \;  for \;  benefit  \; attribute\\ \left({\eta }_{{{t}}_{\mathbb{Y}}}, {\wp }_{{{t}}_{\mathbb{Y}}}, {\zeta }_{{{t}}_{\mathbb{Y}}}\right),\;   for \;  the \;  cost \;  attribute\end{array}\right.$$

where $1\le {t}\le  \underline{{\text{n}}}  , 1\le {\mathbb{Y}}\le {{\mathbbm{f}}}$. But the information given in Table 1, does not required to be normalized.

*Step 2* Using the developed WPFPDMSM operator, we obtained the information given in Table 2, with ${\rm P}\kern-5pt \raisebox{8.5pt}{\rotatebox{180}{J}}_{1}=\left\{{\Xi }_{3},{\Xi }_{4}\right\}$ and ${\rm P}\kern-5pt \raisebox{8.5pt}{\rotatebox{180}{J}}_{2}=\left\{{\Xi }_{1},{\Xi }_{2}\right\}$.

**Table 2:** Aggerated values for $\acute{c} =2$.

	WPFPDMSM operators
${\mathfrak{T}}_{1}$	$\left(\mathrm{0.2082,0.3354,0.4391}\right)$
${\mathfrak{T}}_{2}$	$\left(\mathrm{0.2641,0.2641,0.3354}\right)$
${\mathfrak{T}}_{3}$	$\left(\mathrm{0.1617,0.4391,0.4391}\right)$
${\mathfrak{T}}_{4}$	$\left(\mathrm{0.0858,0.3492,0.4391}\right)$
${\mathfrak{T}}_{5}$	$\left(\mathrm{0.2082,0.2641,0.4391}\right)$

*Step 3* Obtained values ${\mathbb{S}}\left({\widehat{\mathcal{M}}}_{{t}}^{\prime}\right)$ and $\check{{\rm A}}\left({\widehat{\mathcal{M}}}_{{t}}^{\prime}\right)$ of aggregated result ${\widehat{\mathcal{M}}}_{{t}}^{\prime}\left({t}=1, 2,. . ., \underline{{\text{n}}}  \right)$, illustrated in Table 3.

**Table 3:** Ranking Values.

	WPFPDMSM operators
${\mathfrak{T}}_{1}$	$-0.566$
${\mathfrak{T}}_{2}$	$-0.335$
${\mathfrak{T}}_{3}$	$-0.717$
${\mathfrak{T}}_{4}$	$-0.702$
${\mathfrak{T}}_{5}$	$-0.495$

*Step 4* Make a comparison between ${\mathbb{S}}\left({\widehat{\mathcal{M}}}_{1}^{\prime}\right), {\mathbb{S}}\left({\widehat{\mathcal{M}}}_{2}^{\prime}\right),. . . .,{\mathbb{S}}\left({\widehat{\mathcal{M}}}_{ \underline{{\text{n}}}  }^{\prime}\right)$ and $\check{{\rm A}}\left({\widehat{\mathcal{M}}}_{1}^{\prime}\right), \check{{\rm A}}\left({\widehat{\mathcal{M}}}_{2}^{\prime}\right),. . .,\check{{\rm A}}\left({\widehat{\mathcal{M}}}_{ \underline{{\text{n}}}  }^{\prime}\right)$, then ranking all the alternatives to choose the best one, such that

$${\mathfrak{T}}_{2}>{\mathfrak{T}}_{5}>{\mathfrak{T}}_{1}>{\mathfrak{T}}_{4}>{\mathfrak{T}}_{3}$$

From the above, we get the best optimal in the shape of ${\mathfrak{T}}_{2}$.

### Comparative analysis

Comparative analysis is an old technique, used for comparing two or more theories. As a main theme of this analysis, we described the advantages and disadvantages of the invented work with the help of comparison among any two or more theories. For this, we suggested various existing theories, described as.

- AOs for IF information, deduced by Xu^18^.
- MSM operators under IF information, which are interpreted by Shi and Xiao^21^.
- PDMSM operators in the setting of IF information which are deduced by Wang et al.^22^.
- AOs for PFSs which was utilized by Garg^33^.
- AOs based on generalized t-norm and t-conorm for PFSs which were presented by Wei^34^,
- Dombi AOs for PF information which were utilized by Jana et al.^35^

Now reconsider the MADM dilemma solved in Sect. "Numerical example" and apply the existing and diagnosed theories. The final result of the diagnosed and prevailing operators is demonstrated in the shape of Table 4.

**Table 4:** Comparative analysis.

Methods	Score Values	Ranking Values
Xu^18^	⤭⇢⤭⇢⤭	⤭⇢⤭⇢⤭
Shi and Xiao^21^	⤭⇢⤭⇢⤭	⤭⇢⤭⇢⤭
Wang et al.^22^	⤭⇢⤭⇢⤭	⤭⇢⤭⇢⤭
Garg^33^	$\mathrm{0.6797,0.478,0.7804,0.8728,0.5797}$	${\mathfrak{T}}_{4}>{\mathfrak{T}}_{3}>{\mathfrak{T}}_{1}>{\mathfrak{T}}_{5}>{\mathfrak{T}}_{2}$
Wei^34^	$0.2105, -\mathrm{0.016,0.3378,0.5363,0.1119}$	${\mathfrak{T}}_{1}>{\mathfrak{T}}_{4}>{\mathfrak{T}}_{5}>{\mathfrak{T}}_{2}>{\mathfrak{T}}_{3}$
Jana et al.^35^	$\mathrm{0.9985,0.9943,0.0094,0.9958,0.9953}$	${\mathfrak{T}}_{4}>{\mathfrak{T}}_{3}>{\mathfrak{T}}_{1}>{\mathfrak{T}}_{5}>{\mathfrak{T}}_{2}$
Proposed work	$-0.566,-0.335,-0.717,-0.702,-0.495$	${\mathfrak{T}}_{2}>{\mathfrak{T}}_{5}>{\mathfrak{T}}_{1}>{\mathfrak{T}}_{4}>{\mathfrak{T}}_{3}$

The prevailing theories investigated by Xu^18^, Shi and Xiao^21^, and Wang et al.^22^ failed to tackle the PF information because these theories are in the structure of IFS and the notion of IFS merely copes with MD and NMD and can’t consider the AD while, the theory of PF information consider MD, NMD and AD. Thus, the invented theory is more generalized and modified than the prevailing one. The diagnosed AOs can also reduce the notion of IFS by just taking AD equal to zero. Moreover, the information in Table 4 represented that if we choose the information of the invented work, we obtained the finest decision in the form of ${\mathfrak{T}}_{2}$, but if we considered the information in Ref.^33, 35^, we obtained the best decision in the shape of ${\mathfrak{T}}_{4}$ and the information given in Ref.^34^ gives the final result in the shape of ${\mathfrak{T}}_{1}$. Consequently, the anticipated operators are more beneficial and practicable than prevailing operators^33–35^. From the above discussion, it is obvious that the diagnosed AOs have the capability of tackling PF information, IF information, and fuzzy information, and these operators can transform to IFS and FS as.

- In the diagnosed AOs, take AD equal to zero, and the AOs will transform in the model of IFS.
- In the diagnosed AOs, take AD and NMD equal to zero, the AOs will transform in the model of FS.

### Ethical approval

The authors state that this is their original work and it is neither submitted nor under consideration in any other journal simultaneously.

## Conclusion

Using the advantages of the PDMSM operator, where the PDMSM operator has the supremacy which can take justification of interrelationship of distinct characteristics and there is a lot of exploration consequence on it. However, it has not been employed to manage MADM dilemmas represented by picture fuzzy numbers (PFNs). So, in this manuscript, first of all, we deduced AOs such as PDMSM and weighted PDMSM operators in the setting of PF information and called them PFPDMSM and WPFPDMSM operators. We also investigated the related axioms of invented operators such as idempotency, monotonicity, and boundedness. After the development of these AOs, we diagnosed a technique of MADM by employing the deduced PFPDMSM and WPFPDMSM operators in the environment of PF information and we identified the best enterprise resource management scheme that is ${\mathfrak{T}}_{2}$ from the collection of considered 5 schemes with the assistance of the developed technique of MADM within PF information. At the end of the manuscript, we compared the deliberated theory with a few prevailing theories to reveal how the deduced AOs and MADM technique is better and beneficial than certain prevailing theories. We also demonstrated that the diagnosed AOs can transform in the setting of IFS and FS.

In the future, we will revise the fundamental theory of bipolar complex fuzzy set^47, 48^, bipolar complex fuzzy soft set^49^, and complex hesitant fuzzy set^49^ and will try to expand this work in these theories.

## Data Availability

The data will be available on reasonable request to corresponding author.
